# Supplementary material for: Statistical Methods in the Study of Protein Binding and Its Relationship to Drug Bioavailability in Breast Milk
Source: Molecules. 2022 May 26;27(11):3441. doi: 10.3390/molecules27113441 (PMC9182007; doi:10.3390/molecules27113441)
Supplement: Supplementary file 1 [file molecules-27-03441-s001.zip › Wanat Supplementary material.pdf]

Table S1. Biological properties of APIs (n = 129).

API	B1	B2	B3	BB_{vivo}	CNS+/-	LactMed	LLL H	log U/D 7.2	M/P	M/P_{code}	PB	PB_{code}	PhCharge
**acebutolol**	0.09	-0.86	-0.83		0	2	3	-2.2	9.65	4	0.26	5	1
**aceclofenac**	-0.44	-0.66	1.11					-4.6			0.99	1	-1
**acenocumarol**	-1.07	-1.20	0.68		1	1		-2.7			0.987	1	-1
**acetylsalicylic acid**	-4.75	-0.47	-13.67		1	2	3	-3.72	1.08	3	0.995	1	-1
**aciclovir**	-1.71	-1.21	-1.39	-0.836		1	2	-2.09	2.35	4	0.21	5	0
**alprazolam**	0.29	-0.06	1.68	0.019	1	1	3	-4.83	0.18	1	0.8	4	0
**amiodarone**	0.60	-0.14	0.84		1	2		-2.17	8.8	4	0.96	2	1
**amlodipine**	-0.09	-1.05	-1.42		0	1	3	-1.77			0.975	2	1
**amoxilcillin**	-2.23	-1.99	-0.74		0	1	1	-4.76	0.028	1	0.2	5	0
**astemizol**	-0.31	-0.05	-0.31		1			-1.48	4.4	4	0.967	2	1
**atorvastatin**	0.37	-1.24	0.11		0		3	-2.91			0.98	1	-1
**atropine**	-0.34	-0.25	0.19	-0.060	1		3	-2.78			0.18	5	1
**azithromycin**	0.51	-2.33	0.56		0	1	2	-1.39			0.29	5	2
**betaxolol**	0.29	-0.26	-0.17		0	2	3	-2.23	2.75	4	0.5	5	1
**bilastine**	-0.70	-0.71	0.39					-0.61			0.87	3	0
**biperiden**	0.55	0.17	0.69		1			-2.06			0.6	4	1
**bisoprolol**	-0.42	-0.41	0.40		0		3	-2.22			0.3	5	1
**bromazepam**	-0.27	-0.32	0.63		1			-4.19			0.7	4	0
**bromocriptine**	-0.93	-1.34	1.58	-1.100	0	0		-0.76			0.93	3	0
**buspirone**	-0.71	-0.57	-0.08	0.480	1	1	3	-0.52			0.95	2	1
**caffeine**	-0.81	-0.31	-1.20	-0.022	1	2	2	-6.68	0.661	2	0.3	5	0
**capecitabine**	-1.57	-1.38	-0.40		1			-1.79			0.6	4	0
**captopril**	0.18	-0.37	-0.52		1	1	2	-3.61	0.031	1	0.275	5	-1
**carbamazepine**	-4.80	-0.19	-14.67	-0.070	1	2	2	-6.74	0.558	2	0.76	4	0
**carbegoline**	-0.88	-0.60	-0.52		1		4	-2.21			0.41	5	2
**carvedilol**	-0.17	-0.66	-1.69		0		3	-1.04			0.98	1	1
**cefuroxime**	-3.12	-2.64	-1.71		0	0	2	-4.61			0.5	5	-1
**celecoxib**	0.29	-0.70	-0.16		1	1	2	-2.48	0.41	2	0.97	2	0

**celiprolol**	0.07	-0.91	-0.89					-2.3			0.275	5	1
**cephalexin**	-0.06	0.03	-1.32		0	1	1	-4.08	0.012	1	0.14	5	0
**cetrizine**	-0.32	-0.30	0.39	-2.150	1	1	2	-3.74			0.93	3	0
**chloramphenikol**	-1.61	-1.30	-0.79		0	1	4	-3.83	0.6	2	0.55	4	0
**chlorpromazine**	0.24	0.04	1.22	0.950	1	1	3	-2.21	0.4	2	0.9	3	1
**chlortalidone**	-1.70	-1.34	-0.98		1	1	4	-2.37	0.062	1	0.75	4	0
**cimetidine**	-1.64	-1.28	-0.96	-1.420	0	1	1	-0.13	1.7	4	0.17	5	1
**ciprofloxacin**	-0.14	-0.62	-1.59			1	3	-0.36	2.15	4	0.3	5	0
**cisapride**	-0.79	-0.83	0.46		1	1		-0.27	0.045	1	0.975	2	1
**clarithromycin**	-2.57	-2.38	-0.67		0	1	1	-0.96	0.25	1	0.7	4	1
**clindamycin**	0.38	-1.15	0.13		0	1	2	-1.53	0.3	1	0.93	3	1
**clobazam**	-0.67	-0.10	-1.37	0.350	1	1	3	-1.39	0.25	1	0.85	3	-1
**clonidine**	-0.36	-0.04	-0.53	0.150	1	2	3	-0.9	2	4	0.3	5	1
**clorazepate**	-0.95	-1.09	0.71		1	2	3	-1.9	0.2	1	0.975	2	-1
**clozapine**	0.36	0.50	0.22		1		3	-0.13	2.79	4	0.99	1	1
**cyproheptadine**	-0.09	0.10	-0.09		1		3	-1.75			0.975	2	1
**desloratidine**	-0.22	0.15	-0.63		1	1	2	-3.07			0.845	4	1
**diazepam**	0.11	0.02	0.84	0.346	1	2	3	-3.8	0.19	1	0.98	1	0
**digoxin**	-2.46	-2.70	0.67	-1.230	0	1	2	-6.3	0.7	2	0.25	5	0
**dihydroergotamine**	-0.18	-1.34	-1.72		0		4	-0.02			0.93	3	1
**diphenhydramin**	0.27	0.35	0.38		1	1	2	-1.56			0.985	1	1
**doxazosin**	0.46	-1.42	0.41		1	1	4	-0.68			0.98	1	1
**doxycycline**	-2.83	-2.36	-1.60		0	1	3	-0.47	0.34	1	0.9	3	0
**drotaverine**	-0.81	-0.24	-1.42		1			-1.03			0.875	3	1
**enalapril**	-0.93	-0.99	0.49		0	1	2	-4.05	0.02	1	0.55	4	-1
**eplerenone**	-0.73	-0.72	0.32		1		3				0.5	5	0
**escitalopram**	0.27	-0.03	-0.23		1	1	2	-2.37	2.2	4	0.56	4	1
**estradiol benzoate**	-0.97	-1.07	1.44				3				0.95	2	0
**estrone**	-0.87	-1.02	0.77					-3.05			0.95	2	0
**famotidine**	-3.55	-3.26	-1.22		1	1	1	-0.73	1.5	4	0.175	5	1
**fexofenadine**	-0.48	-0.75	1.24	-0.980	0	1	2	-0.27			0.65	4	0

**fluoxetine**	0.43	0.21	0.30		1	2	2	-2.85	0.68	2	0.945	2	1
**flupenthixol**	0.18	-0.29	2.00		1	1		-0.15	1.15	3	0.95	2	1
**furosemide**	-1.84	-1.55	-0.79		0		3	-4.16			0.95	2	-1
**gliclazide**	-0.86	-0.84	0.29		1			-1.13			0.94	3	-1
**haloperidol**	-0.26	-0.10	-0.01	1.340	1	1	3	-0.84	0.64	2	0.92	3	1
**hydrocortisone acetate**	-0.12	-0.42	1.44				2	-5.22			0.95	2	0
**hydroxyzine**	-0.21	-0.03	-0.05	0.285	1	1	1	-0.58			0.93	3	1
**ibuprofen**	0.17	-0.05	1.25	-0.180	1	1	1	-2.79	0	1	0.95	2	-1
**indomethacin**	-1.02	-0.47	-1.38	-1.260	1	1	3	-3.24	0.47	2	0.97	2	-1
**ketoprofen**	0.53	-0.32	0.62		1	1		-2.97			0.99	1	-1
**ketotifen**	-0.54	-0.23	-0.54		1	0	3	-1.64			0.75	4	1
**lamotrigine**	-0.99	-0.90	0.03	0.480	1	2	3	-1.81	0.513	2	0.55	4	0
**levocetrizine**	-0.12	-0.30	1.08		1	1	3	-3.74			0.915	3	0
**levofloxacin**	-0.85	-0.63	-0.36			1	3	-2.01	0.95	3	0.31	5	-1
**loperamide**	0.25	-0.15	1.84	0.770	0		2	-0.56			0.97	2	1
**loratadine**	-0.44	-0.13	-0.52		1	1	1	-2.93	1.2	4	0.98	1	0
**lorazepam**	-0.25	-0.44	1.05	0.440	1	1	3	-3.6	0.205	1	0.87	3	0
**medazepam**	0.56	0.30	1.48		1			-1.02			0.99	1	
**meloxicam**	-2.05	-1.63	-1.25		0		3	-2.7			0.99	1	-1
**methyldopa**	-1.62	-1.11	-1.40		0	1	2	-1.63	0.265	1	0.2	5	0
**mianserin**	0.19	0.44	-0.20	0.990	1	1		-1.06	2.2	4	0.9	3	0
**midazolam**	0.28	0.14	1.04	0.398	1	1	2	-1.17	0.785	2	0.97	2	0
**mirtazapine**	0.03	0.24	-0.09	0.530	1	1	3	-0.9	1.1	3	0.85	3	0
**montelukast**	0.77	-0.58	1.40		1	1	3	-2.44			0.99	1	-1
**naproxen**	-0.10	-0.20	0.83		1	1	4	-2.36	0.16	1	0.99	1	-1
**nebivolol**	-0.02	-0.59	-1.18		1		3	-1.45			0.98	1	1
**olanzapine**	-1.34	-0.80	-1.45			1	2	-0.13	0.38	1	0.175	5	1
**oxazepam**	-0.33	-0.44	0.79	0.580	1	1	3	-3.74	0.1	1	0.85	3	0
**oxybutinin**	0.63	-0.25	0.96		1		3	-1.04			0.92	3	1
**pantoprazole**	-0.15	-0.83	-1.61		0	1	1	-1.13			0.98	1	0

**paracetamol**	-0.79	-0.24	-1.33	-0.463	1	1	1	-2.66	0.84	3	0.25	5	0
**paroxetine**	0.06	-0.09	-0.91		1	1	2	-2.48	0.675	2	0.94	3	1
**pergolide**	-0.17	-0.16	0.48	0.300	1			-0.78			0.9	3	1
**perindopril**	-0.97	-0.99	0.37		0	1	3	-4.05			0.15	5	-1
**phenytoin**	-0.61	-0.38	-0.30	-0.096	1	1	2	-0.88	0.29	1	0.9	3	0
**pindolol**	-0.05	-0.37	-1.28		1	1	3	-2.34			0.4	5	1
**piroxicam**	-1.30	-1.18	-0.13		0	1	2	-2.7	0.02	1	0.99	1	-1
**prednizolone**	-0.96	-0.97	0.34		1		2	-5.26	0.13	1	0.9	3	0
**progesterone**	-0.08	-0.20	0.90			1	3				0.975	2	0
**promazine**	0.16	0.04	0.96	0.880	1			-2.23			0.94	3	1
**promethazine**	-0.36	0.04	-0.77		1		2	-1.78			0.93	3	1
**propafenone**	0.42	-0.39	0.27		0	1	2	-2.11			0.97	2	1
**propranolol**	-0.05	-0.12	0.74	1.110	1	1	2	-2.3	0.396	1	0.9	3	1
**quetiapine**	-0.52	-0.63	0.76		1	1	2	-0.46			0.83	4	1
**quinapril**	0.40	-0.99	0.18		0	1	2	-3.81	0.12	1	0.97	2	-1
**risperidone**	-0.68	-0.44	-0.35	-0.020	1	1	3	-0.87	0.26	1	0.88	3	1
**rizatriptan**	-0.78	-0.40	-0.80	0.780	1		3	-2.29			0.14	5	1
**rosuvastatin**	0.32	-1.71	-0.08		0	1	3	-2.95			0.88	3	-1
**roxitromicin**	0.58	-2.92	0.79		0	1		-0.96	0.04	1	0.96	2	1
**rupatadine**	-0.04	0.08	0.15					-0.25			0.985	1	1
**sertraline**	0.39	0.35	0.18		1	1	2	-2.27	1.85	4	0.98	1	1
**sildenafil**	-0.08	-1.20	-1.40		1	1	3	-1.17			0.96	2	0
**simvastatin**	-0.27	-0.62	1.55		1		3	-6.29			0.95	2	0
**spironolactone**	-0.62	-0.82	1.00		1		2		0.615	2	0.9	3	0
**sulpiride**	-1.42	-1.21	-0.43		1	2	2	-1.77			0.4	5	1
**telmisartan**	0.33	-0.46	3.03		1		4	-3.34			0.995	1	-1
**temazepam**	-0.17	-0.30	0.91		1	1	3	-4.46	0.14	1	0.96	2	0
**theophylline**	-1.09	-0.56	-1.32	-0.341	1	1	3	-1.4	0.697	2	0.4	5	0
**thioridazine**	-0.07	-0.37	1.43	0.285	1		4	-2.64			0.95	2	1
**tolterodine**	0.63	0.17	0.96		1		3	-3.48			0.963	2	1
**trazodone**	-0.06	-0.13	0.74		1	1	2	-0.32	0.53	2	0.92	3	1

**tropicamid**	0.14	-0.31	-0.67		1		3	-1.88			0.45	5	0
**valproic acid**	0.54	-0.05	0.64		1	1	4	-2.38	0.05	1	0.85	3	-1
**valsartan**	-0.93	-1.25	1.28		0		3	-3.64			0.955	2	-1
**venlafaxine**	0.36	0.02	0.05		1	1	3	-2.07	3.32	4	0.27	5	1
**verapamil**	-0.72	-0.48	-0.38	-0.470	1	1	2	-1.77	0.6	2	0.9	3	1
**zolmitriptane**	-0.86	-0.37	-1.17		1		3	-2.32			0.25	5	1
**zolpidem**	-0.29	-0.05	-0.24	-0.480	1	1	3	-0.43	0.15	1	0.92	3	0
**zopiclone**	-0.87	-0.92	0.49		0		2	-0.5	0.51	2	0.45	5	0
**zuclopenthixol**	0.12	-0.29	1.82		1	1	3	-0.17	0.37	1	0.985	1	1

Table S2. Physicochemical properties of APIs (n = 129).

API	a/b/n code[1]	DM	eH	eH-eL	eL[2]	HA	HD	log D	log P	MW[3]	pKa	PSA[4]	Sa[3]	V[3]
**acebutolol**	1	2.65	-9.19	-8.68	-5.11	5	3	0.26	-0.33	3.36	9.4	8.77	6.33	3.29
**aceclofenac**	-1	1.93	-8.85	-8.58	-2.68	5	2	0.44	3.54	3.54	2.6	7.56	4.44	2.84
**acenocumarol**	-1	8.94	-9.74	-8.27	-14.71	7	1	0.34	2.68	3.53	4.5	10.94	4.66	2.96
**acetylsalicylic acid**	-1	5.18	-9.78	-9.08	-6.93	4	1	-1.89	1.30	1.80	3.48	6.36	3.05	1.52
**aciclovir**	0	5.69	-8.76	-8.27	-4.89	8	4	-1.76	-1.45	2.25	5.11	10.98	3.59	1.86
**alprazolam**	0	6.16	-9.51	-9.36	-1.42	4	0	2.5	4.68	3.09	2.37	3.81	2.94	2.69
**amiodarone**	1	5.30	-7.46	-4.11	-33.48	3	0	6.94	3.01	6.45	9.37	4.27	5.21	4.46
**amlodipine**	1	2.88	-8.66	-8.32	-3.35	5	2	2.59	-1.51	4.09	8.97	9.99	5.92	3.62
**amoxilcillin**	-1	4.32	-9.20	-8.87	-3.31	8	5	-2.43	-0.15	3.65	2.44	15.83	4.88	3.07
**astemizol**	1	1.71	-7.83	-7.52	-3.07	5	1	4.08	0.71	4.59	8.68	3.74	6.02	4.22
**atorvastatin**	-1	2.22	-9.00	-8.86	-1.40	5	4	1.11	1.54	5.63	4.29	11.18	6.75	5.21
**atropine**	1	2.48	-9.42	-9.46	0.41	4	1	-0.94	1.71	2.89	9.98	4.98	4.24	2.79
**azithromycin**	1			0.00		13	5	3.18	2.44		8.59	18.01		
**betaxolol**	1	1.20	-8.98	-9.42	4.43	4	2	0.93	1.00	3.07	9.43	5.07	5.89	3.15
**bilastine**	0	2.69	-8.82	-8.70	-1.22	6	2	1.97	2.10	4.64	6.59	7.85	6.65	4.54
**biperiden**	1	2.30	-9.16	-9.48	3.22	2	1	1.68	2.71	3.11	9.26	2.35	4.11	3.20
**bisoprolol**	1	1.93	-9.09	-9.42	3.27	5	2	0.4	2.13	3.25	9.42	6.00	7.05	3.31
**bromazepam**	0	2.87	-9.13	-9.02	-1.07	4	1	1.65	2.59	3.16	11.39	5.44	2.67	2.36
**bromocriptine**	0	4.25	-8.92	-7.96	-9.58	10	3	5.07	4.48	6.69	6.44	11.82	6.06	5.64
**buspirone**	0	4.32	-8.74	-8.58	-1.56	7	0	3.35	1.18	3.86	7.72	6.96	5.19	3.71
**caffeine**	0	3.92	-9.00	-8.47	-5.36	6	0	-0.13	-1.06	1.94	0.52	5.35	3.41	1.68
**capecitabine**	-1	4.97	-9.72	-8.38	-13.37	9	3	-0.73	0.53	3.59	5.41	12.07	5.37	3.04
**captopril**	-1	1.87	-9.35	-9.45	1.05	3	2	-3.15	0.30	2.17	3.59	5.76	3.63	1.92
**carbamazepine**	0	3.25	-9.05	-8.53	-5.19	3	2	2.67	2.10	2.36	13.94	4.63	2.85	2.19
**carbegoline**	1	3.58	-8.39	-8.15	-2.38	7	2	-0.19	0.29	4.24	9.41	7.17	6.41	4.10
**carvedilol**	0	3.15	-8.56	-8.35	-2.09	5	3	3.29	-2.04	4.20	8.24	7.57	5.49	3.97
**cefuroxime**	-1	2.73	-8.76	-7.44	-13.17	4	12	2.91	-2.08	4.24	2.59	19.91	4.26	3.75
**celecoxib**	0	4.09	-9.93	-8.51	-14.21	3	1	4.21	1.02	3.81	9.68	7.80	5.10	2.97

**celiprolol**	1	2.01	-9.24	-8.78	-4.56	5	3	0.22	-0.45	3.65	9.5	9.09	6.62	3.59
**cephalexin**	-1	5.35	-9.59	-8.97	-6.25	5	3	-2.44	-1.31	3.47	3.12	3.25	4.24	2.95
**cetrizine**	-1	3.56	-8.97	-8.75	-2.20	5	1	-1.13	2.11	3.75	3.46	5.30	5.01	3.37
**chloramphenikol**	0	-9.08	-10.34	-9.01	-13.37	7	3	1.02	-0.25	3.23	11.03	11.54	4.50	2.44
**chlorpromazine**	1	7.13	-6.83	-6.90	0.74	2	0	3.26	3.77	3.19	9.41	3.18	3.04	2.62
**chlortalidone**	0	4.04	-9.45	-8.68	-7.73	6	4	-0.74	-0.63	3.19	9.57	11.79	3.79	2.51
**cimetidine**	0	11.01	-8.75	-8.48	-2.76	6	3	-0.25	-0.59	2.52	7.07	11.42	2.94	2.29
**ciprofloxacin**	0	6.93	-8.72	-7.94	-7.85	6	2	-0.85	-1.85	331.35	7.56	7.29	4.25	2.84
**cisapride**	0	1.68	-8.72	-8.54	-1.86	7	3	2.6	2.25	4.66	7.47	8.61	6.66	4.11
**clarithromycin**	0			0.00		14	4	2.33	3.16		8.16	18.29		
**clindamycin**	1			0.00		7	4	0.48	1.59		8.73	10.58		
**clobazam**	0	4.18	-9.21	-8.66	-5.52	4	0	1.59	-1.40	3.01	8.59	4.03	4.02	2.58
**clonidine**	0	3.51	-8.90	-8.48	-4.25	3	2	-0.68	0.28	2.30	8.1	3.64	3.10	1.86
**clorazepate**	-1	3.51	-9.12	-9.01	-1.13	6	4	1.55	2.75	3.15	5.3	10.22	2.91	2.57
**clozapine**	0	1.13	-8.78	-8.51	-2.69	1	0	4.86	1.77	2.87	7.33	0.32	3.81	2.90
**cyproheptadine**	1	1.98	-8.63	-8.83	2.03	3	1	2.44	1.16	2.65	8.95	2.76	3.89	2.66
**desloratidine**	1	3.04	-9.13	-8.83	-2.94	2	1	4.1	0.07	3.11	10.27	2.49	3.75	2.88
**diazepam**	0	3.57	-9.06	-8.96	-1.00	3	0	2.91	3.01	2.85	3.4	3.27	2.80	2.50
**digoxin**	0	5.58	-8.45	-7.28	-11.68	14	6	0.85	2.67	7.81	13.5	20.31	7.66	7.05
**dihydroergotamine**	0	3.90	-8.60	-8.50	-1.05	6	3	2.05	-2.11	4.78	7.22	11.82	5.13	4.33
**diphenhydramin**	1	0.52	-9.18	-9.41	2.36	2	0	2.29	2.09	2.55	8.76	1.25	4.92	2.65
**doxazosin**	0	4.02	-8.67	-7.54	-11.34	9	1	0.6	2.14	4.09	6.52	12.27	4.93	3.46
**doxycycline**	0	11.15	-9.00	-7.85	-11.51	10	7	-3.32	-1.86	4.44	7.67	18.16	4.43	3.68
**drotaverine**	0	3.56	-8.36	-7.99	-3.63	5	1	6.12	-1.51	3.98	6.17	4.90	6.39	3.89
**enalapril**	-1	3.46	-9.37	-9.38	0.20	7	2	-2.35	2.30	3.75	3.15	9.59	4.19	3.48
**eplerenone**		3.20	-10.28	-10.00	-2.81	6	0	1.05	1.96	4.15		7.89	4.43	3.73
**escitalopram**	1	2.57	-9.21	-8.37	-8.44	3	0	0.39	0.87	3.24	9.57	3.63	5.46	3.08
**estradiol benzoate**	0	5.95	-10.11	-9.97	-1.40	4	0	2.51	4.20	3.73		6.04	4.77	3.58
**estrone**	0	5.76	-10.17	-9.95	-2.21	6	1	2.53	2.86	4.02	10.25	9.77	4.73	3.67
**famotidine**	0	1.01	-8.55	-7.97	-5.85	9	8	-1.02	-1.11	3.36	7.93	23.78	4.85	2.69
**fexofenadine**	0	2.61	-8.98	-8.75	-2.28	5	3	2.3	3.81	5.02	6.93	8.10	6.27	4.87

**fluoxetine**	1	4.43	-9.41	-9.02	-3.95	2	1	1.56	1.93	3.09	10.05	2.13	4.99	2.71
**flupenthixol**	0	4.80	-7.61	-6.76	-8.51	3	1	4.42	5.33	4.30	7.05	5.20	5.85	3.65
**furosemide**	-1	3.27	-8.92	-7.90	-10.23	7	4	-0.12	-0.24	3.11	3.04	13.10	4.19	2.42
**gliclazide**	-1	5.31	-9.38	-8.30	-10.80	6	2	-0.28	1.90	3.23	6.07	8.69	4.91	2.86
**haloperidol**	0	2.85	-9.22	-8.75	-4.64	3	1	2.11	1.31	3.76	8.04	4.05	5.29	3.37
**hydrocortisone acetate**	0	4.23	-10.14	-10.06	-0.86	4	0	4.53	4.20	3.73	12.42	6.04	4.77	3.58
**hydroxyzine**	0	1.27	-8.81	-8.75	-0.65	4	1	2	1.23	3.75	6.62	3.59	5.48	3.54
**ibuprofen**	-1	1.91	-9.39	-9.58	1.92	2	1	0.8	3.83	2.06	4.41	3.73	2.52	2.42
**indomethacin**	-1	2.98	-8.69	-8.12	-5.75	5	1	-0.16	-1.43	3.58	3.96	6.36	4.94	3.05
**ketoprofen**	-1	4.50	-9.96	-9.56	-3.99	3	1	-0.25	2.56	2.54	4.23	5.44	4.07	2.36
**ketotifen**	1	4.07	-9.08	-8.07	-10.10	2	0	3.28	0.26	3.09	8.84	4.86	3.89	2.87
**lamotrigine**	0	2.75	-8.22	-7.66	-5.61	5	4	-0.19	1.38	2.56	5.39	9.07	3.15	1.93
**levocetrizine**	-1	1.73	-9.00	-8.83	-1.67	5	1	-1.13	3.48	3.89	3.46	5.30	5.59	3.55
**levofloxacin**	-1	7.21	-8.72	-7.88	-8.37	7	1	0.65	0.62	3.61	5.19	7.33	4.75	3.06
**loperamide**	0	4.65	-8.52	-8.50	-0.23	4	1	3.53	5.01	4.77	7.76	4.38	4.89	4.33
**loratadine**	0	2.90	-9.02	-8.36	-6.59	4	0	5.94	0.30	3.69	4.27	4.24	4.93	3.31
**lorazepam**	0	6.40	-7.77	-7.65	-1.17	4	2	2.47	3.42	3.21	10.8	6.17	2.58	2.53
**medazepam**	0	4.08	-8.34	-8.28	-0.59	2	0	4.42	4.29	3.30	6.18	1.56	2.80	2.49
**meloxicam**	-1	5.35	-7.64	-6.38	-12.59	7	2	-0.09	-1.16	3.51	4.5	13.62	4.03	2.71
**methyldopa**	0	1.56	-9.07	-9.34	2.75	5	5	-2.38	-1.46	2.11	5.57	10.38	3.34	1.85
**mianserin**	1	0.56	-8.66	-8.93	2.68	2	0	2.76	0.94	2.64	8.26	0.65	3.65	2.64
**midazolam**	0	3.03	-9.24	-9.11	-1.28	3	0	3.92	3.41	3.26	6.03	2.53	3.04	2.76
**mirtazapine**	0	1.76	-8.79	-8.81	0.19	3	0	1.97	1.16	2.65	8.1	1.94	3.57	2.60
**montelukast**	-1	3.34	-7.46	-6.83	-6.36	4	2	5.19	4.13	5.88	4.76	7.04	7.27	5.47
**naproxen**	-1	1.92	-9.10	-8.82	-2.79	3	1	0.47	2.99	2.30	4.84	4.65	3.89	2.15
**nebivolol**	1	2.46	-9.05	-8.94	-1.07	5	3	2.4	-1.03	4.05	8.65	7.10	5.05	3.53
**olanzapine**	0	7.55	-9.20	-8.41	-7.85	6	1	-1.48	-1.56	3.28	7.33	8.41	6.19	3.01
**oxazepam**	0	3.38	-9.09	-8.98	-1.06	4	2	2.32	2.91	2.87	10.94	6.17	2.72	2.72
**oxybutinin**	0	1.02	-9.18	-9.11	-0.66	3	1	5.53	3.24	3.57	8.24	4.98	5.82	3.61
**pantoprazole**	0	7.25	-9.09	-8.19	-9.03	6	1	1.5	-1.89	3.83	8.33	8.63	5.08	3.01
**paracetamol**	0	3.28	-8.58	-8.65	0.73	3	2	0.34	-1.32	1.51	9.86	4.93	3.03	1.39

**paroxetine**	1	2.12	-9.00	-9.04	0.34	4	1	1.19	-0.49	3.31	9.68	3.97	4.12	2.99
**pergolide**	0	1.93	-8.35	-8.50	1.51	2	1	2.29	2.29	3.14	7.98	4.43	4.50	3.09
**perindopril**	-1	6.26	-9.78	-10.07	2.88	7	2	-0.01	2.06	3.68	3.15	9.59	5.43	3.52
**phenytoin**	0	2.98	-9.84	-9.55	-2.94	4	2	2.48	0.73	2.52	6.32	5.82	3.32	2.26
**pindolol**	1	3.19	-8.47	-8.53	0.63	3	3	0.19	-1.23	2.48	9.54	5.73	4.60	2.44
**piroxicam**	-1	11.24	-8.63	-8.84	2.05	7	2	-2.14	1.07	3.33	4.5	10.80	2.66	2.50
**prednizolone**	0	3.68	-10.01	-9.54	-4.70	5	3	1.49	2.01	3.46	12.46	9.48	3.96	3.14
**progesterone**		5.88	-9.29	-9.03	-2.60	3	1	6.24	3.12	3.77		4.65	4.54	3.60
**promazine**	1	2.86	-7.54	-7.15	-3.93	2	0	2.67	3.25	2.84	9.43	3.18	2.93	2.53
**promethazine**	1	3.40	-7.83	-7.76	-0.65	2	0	3.2	-0.20	2.84	8.98	3.18	4.36	2.76
**propafenone**	1	2.97	-8.77	-8.59	-1.75	4	2	2.06	1.86	3.29	9.31	5.86	5.36	3.28
**propranolol**	1	1.53	-8.36	-7.79	-5.71	3	2	1.27	2.80	2.59	9.5	4.15	3.09	2.56
**quetiapine**	0	1.57	-8.68	-7.95	-7.24	5	1	1.55	2.84	3.84	6.74	7.36	5.42	3.55
**quinapril**	-1	5.13	-9.50	-9.59	0.91	5	2	0.85	1.69	4.39	3.39	9.59	6.10	4.10
**risperidone**	0	3.42	-8.93	-8.06	-8.76	6	0	2.27	0.63	4.10	8.07	6.19	4.91	3.73
**rizatriptan**	1	1.76	-8.14	-7.45	-6.90	4	1	2.68	-0.27	3.12	9.49	5.91	4.26	2.90
**rosuvastatin**	-1	5.66	-9.19	-8.13	-10.63	8	3	-2.63	1.18	4.82	4.25	14.09	6.89	4.12
**roxitromicin**	0			0.00		16	5	2.9	2.90		8.16	21.69		
**rupatadine**	0	1.61	-8.99	-8.35	-6.43	3	0	5.97	1.63	4.15	6.95	2.90	4.97	3.94
**sertraline**	1	2.87	-9.19	-9.09	-0.94	1	1	2.77	1.68	3.06	9.47	1.20	4.21	2.72
**sildenafil**	0	5.84	-8.91	-9.86	9.47	8	1	2.27	-1.47	4.75	6.03	10.91	6.47	4.20
**simvastatin**	0	5.78	-9.10	-9.33	2.25	5	1	4.41	4.43	4.19	13.49	7.28	5.71	4.10
**spironolactone**		1.67	-9.76	-8.96	-7.98	4	0	3.12	3.33	4.17		8.57	4.94	3.82
**sulpiride**	1	3.65	-7.89	-6.22	-16.74	7	3	-1.12	0.48	3.41	8.97	11.01	5.08	3.02
**telmisartan**	-1	0.71	-8.56	-8.02	-5.48	6	1	4.76	7.39	5.01	3.86	6.31	6.39	4.69
**temazepam**	0	2.48	-9.17	-9.09	-0.83	4	1	2.15	3.15	3.01	11.66	5.29	2.84	2.53
**theophylline**	0	3.47	-9.10	-8.53	-5.77	6	1	-0.2	-1.31	1.80	8.6	6.93	3.02	1.50
**thioridazine**	0	0.98	-7.92	-7.65	-2.68	2	0	3.94	4.18	3.71	9.84	5.71	4.89	3.50
**tolterodine**	1	2.18	-8.95	-9.17	2.20	2	1	2.94	3.24	3.25	10.68	2.35	5.73	3.44
**trazodone**	0	3.41	-8.50	-7.88	-6.21	6	0	1.58	2.80	3.72	7.52	4.24	5.01	3.36
**tropicamid**	0	4.06	-9.68	-9.40	-2.81	3	1	1.15	-0.01	2.84	5.32	5.34	4.15	2.76

**valproic acid**	-1	1.93	-11.19	-12.19	10.01	2	1	0.16	2.61	1.44	4.82	3.73	3.75	5.54
**valsartan**	-1	2.92	-9.69	-9.05	-6.39	8	2	0.05	3.88	4.22	3.56	11.21	5.46	3.92
**venlafaxine**	1	3.25	-9.02	-9.29	2.73	3	1	1.07	1.44	2.77	9.27	3.27	4.44	2.85
**verapamil**	1	5.44	-8.76	-8.85	0.90	6	0	2.33	0.57	4.55	8.97	6.40	7.51	4.53
**zolmitriptane**	1	4.09	-8.73	-8.57	-1.57	5	2	-0.44	-1.01	2.89	9.52	5.74	4.68	2.74
**zolpidem**	0	6.69	-8.37	-8.33	-0.45	4	0	3.02	0.86	3.07	6.77	3.76	3.33	2.94
**zopiclone**	0	5.47	-9.13	-8.95	-1.78	9	0	-42	2.31	3.89	6.7	9.18	3.36	3.86
**zuclopenthixol**	0	2.45	-7.57	-7.12	-4.53	3	1	5.06	4.97	3.97	7.03	5.20	5.58	3.55

1 – code describing the acid-base properties: (-1) - acids; (0) - neutral drugs; (1) - bases

2 – value multiplied by 10

3 – value divided by 100

4 – value divided by 10

Table S3. Chromatographic data and their derivatives obtained from the NP TLC experiment for 129 APIs.

API	C_{NP}*	NP**	NP/C	NP/PSA	R_MNP	R_MNP	R_MNP/C	R_MNP/PSA	R_MNP/PB	R_MNP/logP	R_MNP/B2	NP/B2	NP/PB	NP/logP
**acebutolol**	0.45	0.46	1.02	0.52	0.07	0.09	0.80	0.0008	0.268	-0.2110	-0.081	-0.54	1.77	-1.39
**aceclofenac**	0.89	0.90	1.01	1.19	-0.95	-0.91	1.05	-0.0126	-0.964	-0.2696	1.439	-1.36	0.91	0.25
**acenocumarol**	0.99	0.98	0.99	0.90	-1.69	-2.00	0.85	-0.0154	-1.712	-0.6307	1.404	-0.81	0.99	0.37
**acetylsalicylic acid**	0.89	0.90	1.01	1.41	-0.93	-0.91	1.02	-0.0146	-0.935	-0.7159	1.978	-1.90	0.90	0.69
**aciclovir**	0.73	0.73	0.99	0.66	-0.42	-0.43	0.97	-0.0038	-2.005	0.2903	0.348	-0.60	3.45	-0.50
**alprazolam**	0.85	0.86	1.01	2.25	-0.79	-0.75	1.05	-0.0207	-0.985	-0.1685	12.466	-13.60	1.08	0.18
**amiodarone**	0.55	0.6	1.09	1.41	-0.18	-0.09	2.02	-0.0041	-0.183	-0.0585	1.296	-4.42	0.63	0.20
**amlodipine**	0.56	0.56	1.00	0.56	-0.10	-0.10	1.00	-0.0010	-0.107	0.0694	0.100	-0.53	0.57	-0.37
**amoxilcillin**	0.68	0.68	1.00	0.43	-0.33	-0.33	1.00	-0.0021	-1.637	2.1824	0.165	-0.34	3.40	-4.53
**astemizol**	0.62	0.61	0.98	1.63	-0.19	-0.21	0.91	-0.0052	-0.201	-0.2736	3.791	-11.90	0.63	0.86
**atorvastatin**	0.9	0.9	1.00	0.81	-0.95	-0.95	1.00	-0.0085	-0.974	-0.6196	0.769	-0.72	0.92	0.58
**atropine**	0.27	0.26	0.96	0.52	0.45	0.43	1.05	0.0091	2.524	0.2656	-1.822	-1.04	1.44	0.15
**azithromycin**	0.63	0.63	1.00	0.35	-0.23	-0.23	1.00	-0.0013	-0.797	-0.0947	0.099	-0.27	2.17	0.26
**betaxolol**	0.66	0.65	0.98	1.28	-0.27	-0.29	0.93	-0.0053	-0.538	-0.2688	1.016	-2.46	1.30	0.65
**bilastine**	0.46	0.44	0.96	0.56	0.10	0.07	1.50	0.0013	0.120	0.0499	-0.148	-0.62	0.51	0.21
**biperiden**	0.68	0.65	0.96	2.77	-0.27	-0.33	0.82	-0.0115	-0.448	-0.0992	-1.568	3.79	1.08	0.24
**bisoprolol**	0.67	0.66	0.98	1.09	-0.28	-0.31	0.91	-0.0046	-0.928	-0.1307	0.675	-1.59	2.18	0.31
**bromazepam**	0.87	0.90	1.03	1.65	-0.93	-0.83	1.13	-0.0171	-1.329	-0.3593	2.885	-2.77	1.28	0.35
**bromocriptine**	0.95	0.96	1.01	0.81	-1.38	-1.28	1.08	-0.0117	-1.484	-0.3081	1.027	-0.71	1.03	0.21
**buspirone**	0.57	0.58	1.01	0.83	-0.13	-0.12	1.07	-0.0019	-0.138	-0.1113	0.231	-1.01	0.61	0.49
**caffeine**	0.82	0.82	1.00	1.53	-0.66	-0.66	1.00	-0.0123	-2.195	0.6213	2.130	-2.65	2.73	-0.77
**capecitabine**	0.92	0.92	1.00	0.76	-1.06	-1.06	1.00	-0.0088	-1.768	-2.0013	0.766	-0.66	1.53	1.74
**captopril**	0.77	0.74	0.96	1.28	-0.45	-0.52	0.87	-0.0079	-1.652	-1.5142	1.212	-1.97	2.69	2.47
**carbamazepine**	0.95	0.95	0.99	2.04	-1.24	-1.28	0.97	-0.0267	-1.625	-0.5881	6.357	-4.86	1.24	0.45
**carbegoline**	0.68	0.68	1.00	0.95	-0.33	-0.33	1.00	-0.0046	-0.798	-1.1288	0.546	-1.13	1.66	2.34
**carvedilol**	0.73	0.9	1.23	1.19	-0.95	-0.43	2.21	-0.0126	-0.974	0.4678	1.437	-1.36	0.92	-0.44
**cefuroxime**	0.97	0.98	1.01	0.49	-1.69	-1.51	1.12	-0.0085	-3.380	0.8126	0.641	-0.37	1.96	-0.47
**celecoxib**	0.98	0.99	1.01	1.27	-2.00	-1.69	1.18	-0.0256	-2.057	-1.9565	2.848	-1.41	1.02	0.97
**celiprolol**	0.45	0.46	1.02	0.51	0.07	0.09	0.80	0.0008	0.253	-0.1547	-0.077	-0.51	1.67	-1.02
**cephalexin**	0.9	0.9	1.00	2.77	-0.95	-0.95	1.00	-0.0293	-6.816	0.7284	-35.766	33.73	6.43	-0.69

**cetrizine**	0.64	0.65	1.01	1.22	-0.26	-0.25	1.04	-0.0049	-0.279	-0.1229	0.861	-2.14	0.69	0.31
**chloramphenikol**	0.99	0.97	0.98	0.84	-1.51	-2.00	0.76	-0.0131	-2.745	6.0386	1.162	-0.75	1.76	-3.88
**chlorpromazine**	0.43	0.43	0.99	1.34	0.13	0.12	1.07	0.0041	0.146	0.0348	3.408	11.03	0.47	0.11
**chlortalidone**	0.96	0.96	0.99	0.81	-1.33	-1.38	0.96	-0.0113	-1.769	2.1060	0.991	-0.71	1.27	-1.52
**cimetidine**	0.64	0.67	1.05	0.59	-0.31	-0.25	1.23	-0.0027	-1.809	0.5213	0.240	-0.52	3.94	-1.14
**ciprofloxacin**	0.11	0.11	1.00	0.15	0.91	0.91	1.00	0.0125	3.027	-0.4908	-1.467	-0.18	0.37	-0.06
**cisapride**	0.64	0.63	0.98	0.73	-0.23	-0.25	0.93	-0.0027	-0.237	-0.1027	0.279	-0.76	0.65	0.28
**clarithromycin**	0.99	0.99	0.99	0.54	-1.82	-2.00	0.91	-0.0099	-2.596	-0.5751	0.764	-0.41	1.41	0.31
**clindamycin**	0.69	0.66	0.96	0.62	-0.29	-0.35	0.83	-0.0027	-0.310	-0.1812	0.252	-0.58	0.71	0.42
**clobazam**	0.95	0.95	1.00	2.36	-1.28	-1.28	1.00	-0.0318	-1.504	0.9134	13.161	-9.78	1.12	-0.68
**clonidine**	0.49	0.50	1.02	1.37	0.00	0.02	0.00	0.0000	0.000	0.0000	0.000	-14.00	1.67	1.79
**clorazepate**	0.94	0.94	1.00	0.92	-1.19	-1.19	1.00	-0.0117	-1.226	-0.4345	1.099	-0.86	0.96	0.34
**clozapine**	0.45	0.46	1.01	14.04	0.08	0.09	0.90	0.0242	0.079	0.0443	0.158	0.92	0.46	0.26
**cyproheptadine**	0.45	0.43	0.94	1.54	0.13	0.09	1.51	0.0048	0.135	0.1132	1.251	4.05	0.44	0.37
**desloratidine**	0.36	0.35	0.97	1.40	0.27	0.25	1.08	0.0108	0.318	3.8406	1.813	2.36	0.41	5.00
**diazepam**	0.95	0.95	0.99	2.89	-1.24	-1.28	0.97	-0.0378	-1.260	-0.4103	-50.868	38.92	0.96	0.31
**digoxin**	0.80	0.77	0.96	0.38	-0.51	-0.60	0.85	-0.0025	-2.050	-0.1920	0.190	-0.28	3.06	0.29
**dihydroergotamine**	0.72	0.75	1.04	0.63	-0.48	-0.41	1.16	-0.0040	-0.513	0.2261	0.355	-0.56	0.81	-0.36
**diphenhydramin**	0.42	0.33	0.77	2.61	0.32	0.14	2.26	0.0255	0.322	0.1519	0.913	0.94	0.33	0.16
**doxazosin**	0.8	0.81	1.01	0.66	-0.63	-0.60	1.05	-0.0051	-0.643	-0.2943	0.445	-0.57	0.83	0.38
**doxycycline**	0.06	0.05	0.75	0.02	1.33	1.19	1.11	0.0073	1.474	-0.7133	-0.562	-0.02	0.05	-0.02
**drotaverine**	0.61	0.59	0.97	1.21	-0.16	-0.19	0.81	-0.0032	-0.181	0.1047	0.669	-2.50	0.67	-0.39
**enalapril**	0.33	0.33	0.98	0.34	0.32	0.31	1.03	0.0033	0.577	0.1380	-0.321	-0.33	0.59	0.14
**eplerenone**	0.94	0.94	1.00	1.19	-1.19	-1.19	1.00	-0.0151	-2.390	-0.6097	1.670	-1.31	1.88	0.48
**escitalopram**	0.42	0.42	1.00	1.16	0.14	0.14	1.00	0.0039	0.250	0.1611	-4.227	-12.67	0.75	0.48
**estradiol benzoate**	0.97	0.98	1.01	1.62	-1.69	-1.51	1.12	-0.0280	-1.779	-0.4024	1.583	-0.92	1.03	0.23
**estrone**	0.99	0.99	1.00	1.01	-2.00	-2.00	1.00	-0.0204	-2.101	-0.6978	1.963	-0.97	1.04	0.35
**famotidine**	0.70	0.71	1.01	0.30	-0.39	-0.37	1.06	-0.0016	-2.222	0.3503	0.119	-0.22	4.06	-0.64
**fexofenadine**	0.61	0.60	0.98	0.73	-0.17	-0.19	0.86	-0.0021	-0.257	-0.0438	0.223	-0.79	0.92	0.16
**fluoxetine**	0.68	0.67	0.99	3.15	-0.31	-0.33	0.94	-0.0145	-0.325	-0.1594	-1.487	3.24	0.71	0.35
**flupenthixol**	0.51	0.52	1.02	1.00	-0.03	-0.02	2.00	-0.0007	-0.037	-0.0065	0.122	-1.82	0.55	0.10
**furosemide**	0.92	0.92	1.00	0.70	-1.06	-1.06	1.00	-0.0081	-1.117	4.4196	0.685	-0.59	0.97	-3.83
**gliclazide**	0.95	0.95	1.00	1.09	-1.28	-1.28	1.00	-0.0147	-1.360	-0.6730	1.516	-1.13	1.01	0.50

**haloperidol**	0.66	0.65	0.98	1.59	-0.26	-0.29	0.90	-0.0064	-0.282	-0.1980	2.551	-6.35	0.70	0.49
**hydrocortisone acetate**	0.96	0.96	1.00	1.59	-1.38	-1.38	1.00	-0.0228	-1.453	-0.3286	3.286	-2.29	1.01	0.23
**hydroxyzine**	0.55	0.55	1.00	1.53	-0.09	-0.09	1.00	-0.0024	-0.094	-0.0709	3.108	-19.61	0.59	0.45
**ibuprofen**	0.93	0.90	0.96	2.40	-0.93	-1.12	0.83	-0.0249	-0.980	-0.2430	18.687	-17.97	0.94	0.23
**indomethacin**	0.88	0.90	1.02	1.42	-0.95	-0.87	1.10	-0.0150	-0.984	0.6673	2.028	-1.91	0.93	-0.63
**ketoprofen**	0.89	0.89	1.00	1.64	-0.91	-0.91	1.00	-0.0167	-0.917	-0.3547	2.812	-2.76	0.90	0.35
**ketotifen**	0.34	0.36	1.06	0.74	0.25	0.29	0.87	0.0051	0.333	0.9611	-1.087	-1.57	0.48	1.38
**lamotrigine**	0.89	0.90	1.01	0.99	-0.93	-0.91	1.02	-0.0103	-1.692	-0.6744	1.029	-0.99	1.63	0.65
**levocetrizine**	0.63	0.64	1.02	1.21	-0.25	-0.23	1.08	-0.0047	-0.273	-0.0718	0.830	-2.13	0.70	0.18
**levofloxacin**	0.17	0.17	0.97	0.23	0.70	0.69	1.02	0.0096	2.272	1.1358	-1.125	-0.26	0.53	0.27
**loperamide**	0.68	0.67	0.99	1.53	-0.31	-0.33	0.94	-0.0070	-0.317	-0.0614	2.004	-4.37	0.69	0.13
**loratadine**	0.94	0.94	1.00	2.22	-1.19	-1.19	1.00	-0.0282	-1.219	-3.9833	9.061	-7.13	0.96	3.13
**lorazepam**	0.94	0.94	1.00	1.52	-1.19	-1.19	1.00	-0.0194	-1.374	-0.3494	2.716	-2.14	1.08	0.27
**medazepam**	0.91	0.94	1.03	6.03	-1.19	-1.00	1.19	-0.0766	-1.207	-0.2785	-4.018	3.16	0.95	0.22
**meloxicam**	0.99	0.98	0.99	0.72	-1.69	-2.00	0.85	-0.0124	-1.707	1.4571	1.035	-0.60	0.99	-0.84
**methyldopa**	0.38	0.37	0.96	0.35	0.24	0.21	1.13	0.0023	1.202	-0.1647	-0.216	-0.33	1.83	-0.25
**mianserin**	0.54	0.55	1.01	8.41	-0.08	-0.07	1.13	-0.0121	-0.087	-0.0834	-0.177	1.23	0.61	0.58
**midazolam**	0.82	0.83	1.01	3.27	-0.67	-0.66	1.02	-0.0267	-0.694	-0.1975	-4.709	5.77	0.85	0.24
**mirtazapine**	0.39	0.39	0.99	1.99	0.20	0.19	1.05	0.0105	0.239	0.1754	0.858	1.62	0.45	0.33
**montelukast**	0.95	0.95	1.00	1.35	-1.28	-1.28	1.00	-0.0182	-1.292	-0.3096	2.206	-1.64	0.96	0.23
**naproxen**	0.92	0.89	0.97	1.91	-0.91	-1.06	0.86	-0.0195	-0.917	-0.3037	4.598	-4.51	0.90	0.30
**nebivolol**	0.75	0.75	1.00	1.06	-0.48	-0.48	1.00	-0.0067	-0.487	0.4632	0.811	-1.28	0.77	-0.73
**olanzapine**	0.28	0.29	1.02	0.34	0.40	0.41	0.97	0.0048	2.283	-0.2561	-0.500	-0.36	1.63	-0.18
**oxazepam**	0.93	0.94	1.01	1.52	-1.16	-1.12	1.03	-0.0188	-1.362	-0.3979	2.631	-2.12	1.10	0.32
**oxybutinin**	0.68	0.66	0.97	1.33	-0.29	-0.33	0.88	-0.0058	-0.313	-0.0889	1.155	-2.65	0.72	0.20
**pantoprazole**	0.93	0.92	0.99	1.07	-1.06	-1.12	0.94	-0.0123	-1.082	0.5612	1.271	-1.10	0.94	-0.49
**paracetamol**	0.93	0.94	1.01	1.90	-1.16	-1.12	1.03	-0.0235	-4.632	0.8772	4.779	-3.86	3.74	-0.71
**paroxetine**	0.55	0.56	1.02	1.41	-0.10	-0.09	1.20	-0.0026	-0.111	0.2137	1.183	-6.33	0.60	-1.14
**pergolide**	0.48	0.47	0.98	1.06	0.05	0.03	1.50	0.0012	0.058	0.0228	-0.322	-2.90	0.52	0.21
**perindopril**	0.47	0.46	0.97	0.47	0.08	0.05	1.50	0.0008	0.523	0.0381	-0.079	-0.46	3.03	0.22
**phenytoin**	0.97	0.97	1.00	1.67	-1.51	-1.51	1.00	-0.0259	-1.677	-2.0680	3.929	-2.52	1.08	1.33
**pindolol**	0.53	0.52	0.98	0.91	-0.03	-0.05	0.67	-0.0006	-0.087	0.0283	0.094	-1.41	1.30	-0.42
**piroxicam**	0.98	0.98	1.00	0.91	-1.69	-1.69	1.00	-0.0157	-1.707	-1.5796	1.432	-0.83	0.99	0.92

**prednizolone**	0.94	0.94	0.99	0.99	-1.16	-1.19	0.97	-0.0122	-1.287	-0.5761	1.193	-0.96	1.04	0.47
**progesterone**	0.96	0.96	1.00	2.06	-1.38	-1.38	1.00	-0.0297	-1.416	-0.4424	6.989	-4.86	0.98	0.31
**promazine**	0.39	0.37	0.95	1.16	0.23	0.19	1.19	0.0073	0.246	0.0711	6.000	9.61	0.39	0.11
**promethazine**	0.43	0.42	0.98	1.32	0.14	0.12	1.15	0.0044	0.151	-0.7009	3.639	10.90	0.45	-2.10
**propafenone**	0.57	0.59	1.04	1.01	-0.16	-0.12	1.29	-0.0027	-0.163	-0.0850	0.405	-1.51	0.61	0.32
**propranolol**	0.54	0.50	0.93	1.21	0.00	-0.07	0.00	0.0000	0.000	0.0000	0.000	-4.28	0.56	0.18
**quetiapine**	0.68	0.68	1.00	0.92	-0.33	-0.33	1.00	-0.0044	-0.394	-0.1153	0.519	-1.08	0.82	0.24
**quinapril**	0.44	0.44	1.00	0.46	0.10	0.10	1.00	0.0011	0.108	0.0620	-0.106	-0.45	0.45	0.26
**risperidone**	0.29	0.29	0.98	0.46	0.40	0.39	1.03	0.0064	0.454	0.6341	-0.900	-0.64	0.32	0.45
**rizatriptan**	0.20	0.20	0.98	0.33	0.62	0.60	1.02	0.0104	4.398	-2.2806	-1.544	-0.49	1.39	-0.72
**rosuvastatin**	0.9	0.89	0.99	0.63	-0.91	-0.95	0.95	-0.0064	-1.032	-0.7695	0.532	-0.52	1.01	0.75
**roxitromicin**	0.91	0.92	1.01	0.42	-1.06	-1.00	1.06	-0.0049	-1.105	-0.3658	0.363	-0.31	0.96	0.32
**rupatadine**	0.42	0.43	1.02	1.48	0.12	0.14	0.87	0.0042	0.124	0.0751	1.480	5.20	0.44	0.26
**sertraline**	0.53	0.53	1.00	4.41	-0.05	-0.05	1.00	-0.0043	-0.053	-0.0311	-0.147	1.49	0.54	0.32
**sildenafil**	0.69	0.67	0.97	0.61	-0.31	-0.35	0.89	-0.0028	-0.320	0.2092	0.256	-0.56	0.70	-0.46
**simvastatin**	0.19	0.17	0.89	0.23	0.69	0.63	1.09	0.0095	0.725	0.1554	-1.114	-0.27	0.18	0.04
**spironolactone**	0.98	0.96	0.97	1.11	-1.33	-1.69	0.78	-0.0155	-1.474	-0.3984	1.609	-1.16	1.06	0.29
**sulpiride**	0.27	0.26	0.96	0.24	0.45	0.43	1.05	0.0041	1.136	0.9464	-0.374	-0.21	0.65	0.54
**telmisartan**	0.90	0.90	0.99	1.42	-0.93	-0.95	0.98	-0.0148	-0.935	-0.1259	2.013	-1.94	0.90	0.12
**temazepam**	0.94	0.94	0.99	1.77	-1.16	-1.19	0.97	-0.0219	-1.206	-0.3676	3.867	-3.12	0.97	0.30
**theophylline**	0.86	0.87	1.01	1.25	-0.81	-0.79	1.02	-0.0116	-2.017	0.6158	1.436	-1.54	2.16	-0.66
**thioridazine**	0.47	0.43	0.91	0.75	0.12	0.05	2.35	0.0021	0.129	0.0293	-0.334	-1.17	0.45	0.10
**tolterodine**	0.1	0.08	0.80	0.34	1.06	0.95	1.11	0.0452	1.101	0.3274	6.186	0.47	0.08	0.02
**trazodone**	0.71	0.72	1.01	1.69	-0.40	-0.39	1.03	-0.0094	-0.434	-0.1427	3.044	-5.45	0.78	0.26
**tropicamid**	0.85	0.86	1.01	1.61	-0.79	-0.75	1.05	-0.0148	-1.752	78.8370	2.561	-2.79	1.91	-86.00
**valproic acid**	0.69	0.67	0.97	1.80	-0.31	-0.35	0.89	-0.0082	-0.362	-0.1178	6.176	-13.45	0.79	0.26
**valsartan**	0.89	0.91	1.02	0.81	-0.98	-0.91	1.08	-0.0087	-1.025	-0.2523	0.786	-0.73	0.95	0.23
**venlafaxine**	0.36	0.37	1.03	1.13	0.23	0.25	0.93	0.0071	0.856	0.1605	9.712	15.55	1.37	0.26
**verapamil**	0.54	0.53	0.97	0.82	-0.04	-0.07	0.62	-0.0007	-0.048	-0.0763	0.091	-1.10	0.58	0.92
**zolmitriptane**	0.31	0.29	0.94	0.51	0.39	0.35	1.12	0.0068	1.555	-0.3850	-1.049	-0.78	1.16	-0.29
**zolpidem**	0.86	0.88	1.02	2.34	-0.87	-0.79	1.10	-0.0230	-0.941	-1.0062	15.802	-16.07	0.96	1.02
**zopiclone**	0.96	0.96	1.00	1.05	-1.38	-1.38	1.00	-0.0150	-3.067	-0.5975	1.498	-1.04	2.13	0.42
**zuclopenthixol**	0.47	0.47	1.00	0.90	0.05	0.05	1.00	0.0010	0.053	0.0105	-0.183	-1.65	0.48	0.09

* R_f value from the non-impregnated NP plate (control)

** R_f value from the NP plate impregnated with 2 mg/mL BSA solution

Table S4. Chromatographic data and their derivatives obtained from the RP TLC experiment for 129 APIs.

API	C_{RP}*	RP**	RP/C	RP/PSA	R_MRP	R_MRP	R_MRP/C	R_MRP/PSA	R_MRP/PB	R_MRP/logP	R_MRP/B2	RP/B2	RP/PB	RP/logP
**acebutolol**	0.78	0.82	1.05	0.94	-0.55	-0.66	1.20	-0.01	-2.533	1.996	0.770	-0.96	3.15	-2.48
**aceclofenac**	0.93	0.93	1.00	1.23	-1.12	-1.12	1.00	-0.01	-1.135	-0.317	1.694	-1.40	0.94	0.26
**acenocumarol**	0.93	0.92	0.99	0.84	-1.12	-1.06	0.94	-0.01	-1.075	-0.396	0.881	-0.76	0.93	0.34
**acetylsalicylic acid**	0.98	0.99	1.01	1.56	-1.69	-2.00	1.18	-0.03	-2.006	-1.535	4.241	-2.10	0.99	0.76
**aciclovir**	0.99	0.98	0.99	0.89	-2.00	-1.69	0.85	-0.02	-8.049	1.166	1.397	-0.81	4.67	-0.68
**alprazolam**	0.80	0.78	0.98	2.05	-0.60	-0.55	0.91	-0.01	-0.687	-0.117	8.692	-12.33	0.98	0.17
**amiodarone**	0.33	0.34	1.03	0.80	0.31	0.29	0.94	0.01	0.300	0.096	-2.120	-2.50	0.35	0.11
**amlodipine**	0.61	0.68	1.11	0.68	-0.19	-0.33	1.69	0.00	-0.336	0.217	0.311	-0.65	0.70	-0.45
**amoxilcillin**	0.99	0.98	0.99	0.62	-2.00	-1.69	0.85	-0.01	-8.451	11.268	0.851	-0.49	4.90	-6.53
**astemizol**	0.65	0.62	0.95	1.66	-0.27	-0.21	0.79	-0.01	-0.220	-0.299	4.149	-12.10	0.64	0.87
**atorvastatin**	0.80	0.89	1.11	0.80	-0.60	-0.91	1.51	-0.01	-0.927	-0.590	0.731	-0.72	0.91	0.58
**atropine**	0.73	0.72	0.99	1.45	-0.43	-0.41	0.95	-0.01	-2.279	-0.240	1.645	-2.89	4.00	0.42
**azithromycin**	0.24	0.24	1.00	0.13	0.50	0.50	1.00	0.00	1.726	0.205	-0.214	-0.10	0.83	0.10
**betaxolol**	0.27	0.25	0.93	0.49	0.43	0.48	1.10	0.01	0.954	0.477	-1.804	-0.95	0.50	0.25
**bilastine**	0.76	0.72	0.95	0.92	-0.50	-0.41	0.82	-0.01	-0.471	-0.195	0.579	-1.02	0.83	0.34
**biperiden**	0.87	0.85	0.98	3.62	-0.83	-0.75	0.91	-0.03	-1.256	-0.278	-4.393	4.96	1.42	0.31
**bisoprolol**	0.57	0.56	0.98	0.93	-0.12	-0.10	0.86	0.00	-0.349	-0.049	0.254	-1.36	1.87	0.26
**bromazepam**	0.83	0.81	0.98	1.49	-0.69	-0.63	0.91	-0.01	-0.900	-0.243	1.952	-2.51	1.16	0.31
**bromocriptine**	0.69	0.70	1.01	0.59	-0.35	-0.37	1.06	0.00	-0.396	-0.082	0.274	-0.52	0.75	0.16
**buspirone**	0.70	0.69	0.99	0.99	-0.37	-0.35	0.94	0.00	-0.366	-0.294	0.613	-1.22	0.73	0.58
**caffeine**	0.91	0.90	0.99	1.68	-1.00	-0.95	0.95	-0.02	-3.181	0.900	3.087	-2.91	3.00	-0.85
**capecitabine**	0.90	0.89	0.99	0.74	-0.95	-0.91	0.95	-0.01	-1.513	-1.713	0.656	-0.64	1.48	1.68
**captopril**	0.99	0.99	1.00	1.72	-2.00	-2.00	1.00	-0.03	-7.257	-6.652	5.325	-2.64	3.60	3.30
**carbamazepine**	0.83	0.84	1.01	1.81	-0.69	-0.72	1.05	-0.02	-0.948	-0.343	3.707	-4.32	1.11	0.40
**carbegoline**	0.68	0.68	1.00	0.95	-0.33	-0.33	1.00	0.00	-0.798	-1.129	0.546	-1.13	1.66	2.34
**carvedilol**	0.59	0.61	1.03	0.81	-0.16	-0.19	1.23	0.00	-0.198	0.095	0.292	-0.92	0.62	-0.30
**cefuroxime**	0.88	0.87	0.99	0.44	-0.87	-0.83	0.95	0.00	-1.651	0.397	0.313	-0.33	1.74	-0.42
**celecoxib**	0.75	0.75	1.00	0.96	-0.48	-0.48	1.00	-0.01	-0.492	-0.468	0.681	-1.07	0.77	0.74
**celiprolol**	0.75	0.79	1.05	0.87	-0.48	-0.58	1.21	-0.01	-2.092	1.279	0.634	-0.87	2.87	-1.76
**cephalexin**	0.99	0.99	1.00	3.04	-2.00	-2.00	1.00	-0.06	-14.255	1.523	-74.799	37.11	7.07	-0.76

**cetrizine**	0.83	0.82	0.99	1.55	-0.69	-0.66	0.96	-0.01	-0.708	-0.312	2.187	-2.72	0.88	0.39
**chloramphenikol**	0.89	0.89	1.00	0.77	-0.91	-0.91	1.00	-0.01	-1.651	3.632	0.699	-0.69	1.62	-3.56
**chlorpromazine**	0.49	0.50	1.02	1.57	0.02	0.00	0.00	0.00	0.000	0.000	0.000	12.98	0.56	0.13
**chlortalidone**	0.91	0.92	1.01	0.78	-1.00	-1.06	1.06	-0.01	-1.414	1.684	0.792	-0.69	1.23	-1.46
**cimetidine**	0.88	0.87	0.99	0.76	-0.87	-0.83	0.95	-0.01	-4.856	1.399	0.645	-0.68	5.12	-1.47
**ciprofloxacin**	0.38	0.21	0.55	0.29	0.21	0.58	2.71	0.01	1.918	-0.311	-0.929	-0.34	0.70	-0.11
**cisapride**	0.65	0.62	0.95	0.72	-0.27	-0.21	0.79	0.00	-0.218	-0.094	0.256	-0.75	0.64	0.28
**clarithromycin**	0.83	0.78	0.94	0.43	-0.69	-0.55	0.80	0.00	-0.785	-0.174	0.231	-0.33	1.11	0.25
**clindamycin**	0.25	0.25	1.00	0.24	0.48	0.48	1.00	0.00	0.513	0.300	-0.417	-0.22	0.27	0.16
**clobazam**	0.79	0.80	1.01	1.99	-0.58	-0.60	1.05	-0.01	-0.708	0.430	6.197	-8.23	0.94	-0.57
**clonidine**	0.73	0.68	0.93	1.87	-0.43	-0.33	0.76	-0.01	-1.091	-1.169	9.165	-19.04	2.27	2.43
**clorazepate**	0.81	0.81	1.00	0.79	-0.63	-0.63	1.00	-0.01	-0.646	-0.229	0.579	-0.74	0.83	0.29
**clozapine**	0.57	0.57	1.00	17.59	-0.12	-0.12	1.00	-0.04	-0.124	-0.069	-0.247	1.15	0.58	0.32
**cyproheptadine**	0.45	0.47	1.04	1.70	0.09	0.05	0.60	0.00	0.054	0.045	0.497	4.48	0.48	0.41
**desloratidine**	0.58	0.58	1.00	2.33	-0.14	-0.14	1.00	-0.01	-0.166	-2.003	-0.945	3.91	0.69	8.29
**diazepam**	0.77	0.77	1.00	2.36	-0.52	-0.52	1.00	-0.02	-0.535	-0.174	-21.613	31.71	0.79	0.26
**digoxin**	0.84	0.85	1.01	0.42	-0.72	-0.75	1.05	0.00	-3.013	-0.282	0.279	-0.31	3.40	0.32
**dihydroergotamine**	0.78	0.78	1.00	0.66	-0.55	-0.55	1.00	0.00	-0.591	0.261	0.409	-0.58	0.84	-0.37
**diphenhydramin**	0.58	0.54	0.93	4.33	-0.14	-0.07	0.50	-0.01	-0.071	-0.033	-0.200	1.55	0.55	0.26
**doxazosin**	0.75	0.73	0.97	0.59	-0.48	-0.43	0.91	0.00	-0.441	-0.202	0.305	-0.52	0.74	0.34
**doxycycline**	0.08	0.09	1.13	0.05	1.06	1.00	0.95	0.01	1.116	-0.540	-0.426	-0.04	0.10	-0.05
**drotaverine**	0.55	0.58	1.05	1.18	-0.09	-0.14	1.61	0.00	-0.160	0.093	0.593	-2.46	0.66	-0.38
**enalapril**	0.60	0.58	0.97	0.60	-0.18	-0.14	0.80	0.00	-0.255	-0.061	0.142	-0.59	1.05	0.25
**eplerenone**	0.84	0.85	1.01	1.08	-0.72	-0.75	1.05	-0.01	-1.507	-0.384	1.053	-1.19	1.70	0.43
**escitalopram**	0.55	0.52	0.95	1.43	-0.09	-0.03	0.40	0.00	-0.062	-0.040	1.048	-15.68	0.93	0.60
**estradiol benzoate**	0.63	0.62	0.98	1.03	-0.23	-0.21	0.92	0.00	-0.224	-0.051	0.199	-0.58	0.65	0.15
**estrone**	0.79	0.77	0.97	0.79	-0.58	-0.52	0.91	-0.01	-0.552	-0.183	0.516	-0.76	0.81	0.27
**famotidine**	0.98	0.99	1.01	0.42	-1.69	-2.00	1.18	-0.01	-11.404	1.798	0.613	-0.30	5.66	-0.89
**fexofenadine**	0.85	0.86	1.01	1.06	-0.75	-0.79	1.05	-0.01	-1.213	-0.207	1.053	-1.15	1.32	0.23
**fluoxetine**	0.54	0.73	1.35	3.43	-0.07	-0.43	6.20	-0.02	-0.457	-0.224	-2.088	3.53	0.77	0.38
**flupenthixol**	0.66	0.66	1.00	1.27	-0.29	-0.29	1.00	-0.01	-0.303	-0.054	1.010	-2.31	0.69	0.12
**furosemide**	0.96	0.96	1.00	0.73	-1.38	-1.38	1.00	-0.01	-1.453	5.751	0.891	-0.62	1.01	-4.00
**gliclazide**	0.83	0.80	0.96	0.92	-0.69	-0.60	0.87	-0.01	-0.640	-0.317	0.714	-0.95	0.85	0.42

**haloperidol**	0.61	0.64	1.05	1.58	-0.19	-0.25	1.29	-0.01	-0.272	-0.191	2.458	-6.30	0.70	0.49
**hydrocortisone acetate**	0.82	0.82	1.00	1.36	-0.66	-0.66	1.00	-0.01	-0.693	-0.157	1.568	-1.95	0.86	0.20
**hydroxyzine**	0.62	0.59	0.95	1.64	-0.21	-0.16	0.74	0.00	-0.170	-0.129	5.637	-21.04	0.63	0.48
**ibuprofen**	0.83	0.81	0.98	2.17	-0.69	-0.63	0.91	-0.02	-0.663	-0.164	12.645	-16.27	0.85	0.21
**indomethacin**	0.88	0.87	0.99	1.37	-0.87	-0.83	0.95	-0.01	-0.851	0.577	1.754	-1.85	0.90	-0.61
**ketoprofen**	0.91	0.86	0.95	1.58	-1.00	-0.79	0.78	-0.01	-0.796	-0.308	2.441	-2.66	0.87	0.34
**ketotifen**	0.75	0.74	0.99	1.52	-0.48	-0.45	0.95	-0.01	-0.606	-1.747	1.977	-3.22	0.99	2.85
**lamotrigine**	0.87	0.86	0.99	0.95	-0.83	-0.79	0.95	-0.01	-1.433	-0.571	0.872	-0.95	1.56	0.62
**levocetrizine**	0.83	0.83	1.00	1.57	-0.69	-0.69	1.00	-0.01	-0.753	-0.198	2.287	-2.76	0.91	0.24
**levofloxacin**	0.27	0.30	1.11	0.41	0.43	0.37	0.85	0.01	1.187	0.594	-0.588	-0.48	0.97	0.48
**loperamide**	0.75	0.77	1.03	1.76	-0.48	-0.52	1.10	-0.01	-0.541	-0.105	3.419	-5.02	0.79	0.15
**loratadine**	0.68	0.68	1.00	1.60	-0.33	-0.33	1.00	-0.01	-0.334	-1.091	2.482	-5.16	0.69	2.27
**lorazepam**	0.83	0.83	1.00	1.35	-0.69	-0.69	1.00	-0.01	-0.792	-0.201	1.565	-1.89	0.95	0.24
**medazepam**	0.66	0.65	0.98	4.17	-0.29	-0.27	0.93	-0.02	-0.272	-0.063	-0.904	2.19	0.66	0.15
**meloxicam**	0.95	0.95	1.00	0.70	-1.28	-1.28	1.00	-0.01	-1.292	1.102	0.783	-0.58	0.96	-0.82
**methyldopa**	0.85	0.86	1.01	0.83	-0.75	-0.79	1.05	-0.01	-3.942	0.540	0.708	-0.77	4.30	-0.59
**mianserin**	0.55	0.55	1.00	8.49	-0.09	-0.09	1.00	-0.01	-0.097	-0.093	-0.197	1.24	0.61	0.59
**midazolam**	0.66	0.66	1.00	2.61	-0.29	-0.29	1.00	-0.01	-0.297	-0.084	-2.014	4.62	0.68	0.19
**mirtazapine**	0.58	0.58	1.00	2.99	-0.14	-0.14	1.00	-0.01	-0.165	-0.121	-0.591	2.45	0.68	0.50
**montelukast**	0.70	0.69	0.99	0.98	-0.37	-0.35	0.94	0.00	-0.351	-0.084	0.599	-1.19	0.70	0.17
**naproxen**	0.88	0.87	0.99	1.87	-0.87	-0.83	0.95	-0.02	-0.834	-0.276	4.181	-4.41	0.88	0.29
**nebivolol**	0.40	0.40	1.00	0.56	0.18	0.18	1.00	0.00	0.180	-0.171	-0.299	-0.68	0.41	-0.39
**olanzapine**	0.53	0.50	0.94	0.59	-0.05	0.00	0.00	0.00	0.000	0.000	0.000	-0.63	2.86	-0.32
**oxazepam**	0.83	0.84	1.01	1.36	-0.69	-0.72	1.05	-0.01	-0.847	-0.247	1.637	-1.91	0.99	0.29
**oxybutinin**	0.29	0.24	0.83	0.48	0.39	0.50	1.29	0.01	0.544	0.155	-2.008	-0.96	0.26	0.07
**pantoprazole**	0.85	0.83	0.98	0.96	-0.75	-0.69	0.91	-0.01	-0.703	0.364	0.825	-0.99	0.85	-0.44
**paracetamol**	0.92	0.92	1.00	1.86	-1.06	-1.06	1.00	-0.02	-4.243	0.804	4.378	-3.80	3.68	-0.70
**paroxetine**	0.60	0.67	1.12	1.69	-0.18	-0.31	1.75	-0.01	-0.327	0.628	3.474	-7.57	0.71	-1.37
**pergolide**	0.55	0.53	0.96	1.20	-0.09	-0.05	0.60	0.00	-0.058	-0.023	0.322	-3.27	0.59	0.23
**perindopril**	0.57	0.58	1.02	0.60	-0.12	-0.14	1.15	0.00	-0.935	-0.068	0.142	-0.59	3.87	0.28
**phenytoin**	0.87	0.86	0.99	1.48	-0.83	-0.79	0.95	-0.01	-0.876	-1.080	2.052	-2.24	0.96	1.18
**pindolol**	0.76	0.78	1.03	1.36	-0.50	-0.55	1.10	-0.01	-1.374	0.447	1.488	-2.11	1.95	-0.63
**piroxicam**	0.58	0.60	1.03	0.56	-0.14	-0.18	1.26	0.00	-0.178	-0.165	0.149	-0.51	0.61	0.56

**prednizolone**	0.87	0.88	1.01	0.93	-0.83	-0.87	1.05	-0.01	-0.961	-0.430	0.892	-0.91	0.98	0.44
**progesterone**	0.71	0.68	0.96	1.46	-0.39	-0.33	0.84	-0.01	-0.336	-0.105	1.658	-3.44	0.70	0.22
**promazine**	0.51	0.51	1.00	1.60	-0.02	-0.02	1.00	0.00	-0.018	-0.005	-0.451	13.24	0.54	0.16
**promethazine**	0.49	0.47	0.96	1.48	0.02	0.05	3.00	0.00	0.056	-0.261	1.355	12.20	0.51	-2.35
**propafenone**	0.56	0.61	1.09	1.04	-0.10	-0.19	1.85	0.00	-0.200	-0.104	0.498	-1.56	0.63	0.33
**propranolol**	0.64	0.63	0.98	1.52	-0.25	-0.23	0.93	-0.01	-0.257	-0.083	1.978	-5.39	0.70	0.23
**quetiapine**	0.73	0.69	0.95	0.94	-0.43	-0.35	0.80	0.00	-0.419	-0.122	0.551	-1.09	0.83	0.24
**quinapril**	0.57	0.56	0.98	0.58	-0.12	-0.10	0.86	0.00	-0.108	-0.062	0.106	-0.57	0.58	0.33
**risperidone**	0.62	0.58	0.94	0.94	-0.21	-0.14	0.66	0.00	-0.159	-0.223	0.316	-1.31	0.66	0.92
**rizatriptan**	0.72	0.77	1.07	1.30	-0.41	-0.52	1.28	-0.01	-3.748	1.944	1.316	-1.93	5.50	-2.85
**rosuvastatin**	0.92	0.88	0.96	0.62	-1.06	-0.87	0.82	-0.01	-0.983	-0.733	0.507	-0.52	1.00	0.75
**roxitromicin**	0.80	0.79	0.99	0.36	-0.60	-0.58	0.96	0.00	-0.599	-0.198	0.197	-0.27	0.82	0.27
**rupatadine**	0.55	0.52	0.95	1.79	-0.09	-0.03	0.40	0.00	-0.035	-0.021	-0.420	6.29	0.53	0.32
**sertraline**	0.49	0.48	0.98	3.99	0.02	0.03	2.00	0.00	0.035	0.021	0.098	1.35	0.49	0.29
**sildenafil**	0.77	0.76	0.99	0.70	-0.52	-0.50	0.95	0.00	-0.521	0.341	0.417	-0.63	0.79	-0.52
**simvastatin**	0.66	0.65	0.98	0.89	-0.29	-0.27	0.93	0.00	-0.283	-0.061	0.435	-1.05	0.68	0.15
**spironolactone**	0.78	0.77	0.99	0.90	-0.55	-0.52	0.95	-0.01	-0.583	-0.158	0.636	-0.93	0.86	0.23
**sulpiride**	0.85	0.86	1.01	0.78	-0.75	-0.79	1.05	-0.01	-1.971	-1.642	0.649	-0.71	2.15	1.79
**telmisartan**	0.87	0.85	0.98	1.35	-0.83	-0.75	0.91	-0.01	-0.757	-0.102	1.630	-1.84	0.85	0.12
**temazepam**	0.79	0.79	1.00	1.49	-0.58	-0.58	1.00	-0.01	-0.599	-0.183	1.922	-2.64	0.82	0.25
**theophylline**	0.94	0.93	0.99	1.34	-1.19	-1.12	0.94	-0.02	-2.808	0.858	2.000	-1.66	2.33	-0.71
**thioridazine**	0.40	0.40	1.00	0.70	0.18	0.18	1.00	0.00	0.185	0.042	-0.481	-1.09	0.42	0.10
**tolterodine**	0.45	0.43	0.96	1.83	0.09	0.12	1.40	0.01	0.127	0.038	0.714	2.51	0.45	0.13
**trazodone**	0.76	0.74	0.97	1.75	-0.50	-0.45	0.91	-0.01	-0.494	-0.162	3.461	-5.64	0.80	0.26
**tropicamid**	0.83	0.83	1.00	1.55	-0.69	-0.69	1.00	-0.01	-1.530	68.863	2.237	-2.70	1.84	-83.00
**valproic acid**	0.04	0.04	1.00	0.11	1.38	1.38	1.00	0.04	1.624	0.529	-27.715	-0.80	0.05	0.02
**valsartan**	0.97	0.96	0.99	0.86	-1.51	-1.38	0.91	-0.01	-1.445	-0.356	1.108	-0.77	1.01	0.25
**venlafaxine**	0.61	0.65	1.07	1.99	-0.19	-0.27	1.38	-0.01	-0.996	-0.187	-11.296	27.31	2.41	0.45
**verapamil**	0.86	0.86	1.00	1.34	-0.79	-0.79	1.00	-0.01	-0.876	-1.383	1.656	-1.81	0.96	1.51
**zolmitriptane**	0.86	0.86	1.00	1.50	-0.79	-0.79	1.00	-0.01	-3.153	0.781	2.126	-2.32	3.44	-0.85
**zolpidem**	0.76	0.77	1.01	2.05	-0.50	-0.52	1.05	-0.01	-0.570	-0.610	9.583	-14.06	0.84	0.90
**zopiclone**	0.84	0.83	0.99	0.90	-0.72	-0.69	0.96	-0.01	-1.530	-0.298	0.748	-0.90	1.84	0.36
**zuclopenthixol**	0.62	0.60	0.97	1.15	-0.21	-0.18	0.83	0.00	-0.179	-0.035	0.618	-2.10	0.61	0.12

* R_f value from the non-impregnated RP plate (control)

** R_f value from the RP plate impregnated with 2 mg/mL BSA solution

Table S5. Chromatographic data obtained for 129 compounds from $HPLC_{HSA}$ and $HPLC_{IAM}$ experiments and their derivatives.

API	k_{HSA}	log k_{HSA}	log k_{HSA} /B2	log k_{HSA} /log P	log k_{HSA} /PB	log k_{HSA} /PSA	k_{IAM}	log k_{IAM}	log k_{IAM} /B2	log k_{IAM} /log P	log k_{IAM} /PB	log k_{IAM} /PSA
**acebutolol**	1.13	0.05	-0.06	-0.16	0.20	0.001	5.06	0.70	-0.82	-2.13	2.71	0.01
**aceclofenac**	55.89	1.75	-2.64	0.49	1.76	0.023	2.63	0.42	-0.63	0.12	0.42	0.01
**acenocumarol**	18.58	1.27	-1.05	0.47	1.29	0.012	0.53	-0.27	0.23	-0.10	-0.28	0.00
**acetylsalicylic acid**	0.31	-0.51	1.08	-0.39	-0.51	-0.008	0.03	-1.52	3.22	-1.17	-1.52	-0.02
**aciclovir**	0.19	-0.72	0.60	0.50	-3.43	-0.007	0.09	-1.04	0.86	0.72	-4.94	-0.01
**alprazolam**	0.89	-0.05	0.80	-0.01	-0.06	-0.001	3.14	0.50	-7.85	0.11	0.62	0.01
**amiodarone**	124.01	2.09	-15.41	0.70	2.18	0.049	99.63	2.00	-14.71	0.66	2.08	0.05
**amlodipine**	5.45	0.74	-0.70	-0.49	0.75	0.007	98.90	2.00	-1.90	-1.32	2.05	0.02
**amoxilcillin**	0.94	-0.03	0.01	0.18	-0.13	0.000	0.05	-1.32	0.66	8.80	-6.60	-0.01
**astemizol**	13.32	1.12	-21.95	1.58	1.16	0.030	26.27	1.42	-27.70	2.00	1.47	0.04
**atorvastatin**	3.48	0.54	-0.44	0.35	0.55	0.005	8.28	0.92	-0.74	0.60	0.94	0.01
**atropine**	1.23	0.09	-0.36	0.05	0.50	0.002	3.91	0.59	-2.38	0.35	3.29	0.01
**azithromycin**	0.23	-0.64	0.27	-0.26	-2.20	-0.004	0.06	-1.20	0.51	-0.49	-4.14	-0.01
**betaxolol**	1.59	0.20	-0.76	0.20	0.40	0.004	15.60	1.19	-4.51	1.19	2.39	0.02
**bilastine**	0.67	-0.17	0.25	-0.08	-0.20	-0.002	0.55	-0.26	0.36	-0.12	-0.30	0.00
**biperiden**	0.58	-0.24	-1.38	-0.09	-0.39	-0.010	41.47	1.62	9.43	0.60	2.70	0.07
**bisoprolol**	1.07	0.03	-0.07	0.01	0.10	0.000	6.74	0.83	-2.01	0.39	2.76	0.01
**bromazepam**	0.63	-0.20	0.62	-0.08	-0.29	-0.004	2.21	0.34	-1.07	0.13	0.49	0.01
**bromocriptine**	8.09	0.91	-0.68	0.20	0.98	0.008	12.69	1.10	-0.82	0.25	1.19	0.01
**buspirone**	0.89	-0.05	0.09	-0.04	-0.05	-0.001	7.00	0.84	-1.49	0.72	0.89	0.01
**caffeine**	0.23	-0.64	2.06	0.60	-2.13	-0.012	0.18	-0.75	2.44	0.71	-2.51	-0.01
**capecitabine**	0.3	-0.52	0.38	-0.99	-0.87	-0.004	1.11	0.04	-0.03	0.08	0.07	0.00
**captopril**	0.15	-0.82	2.20	-2.75	-3.00	-0.014	0.01	-2.19	5.83	-7.28	-7.95	-0.04
**carbamazepine**	1.09	0.04	-0.19	0.02	0.05	0.001	1.50	0.18	-0.91	0.08	0.23	0.00
**carbegoline**	3.23	0.51	-0.85	1.76	1.24	0.007	17.05	1.23	-2.05	4.25	3.00	0.02
**carvedilol**	14.5	1.16	-1.75	-0.57	1.19	0.015	0.00					
**cefuroxime**	0.23	-0.64	0.24	0.31	-1.28	-0.003	1.69	0.23	-0.09	-0.11	0.45	0.00

**celecoxib**	13.42	1.13	-1.61	1.11	1.16	0.014	50.01	1.70	-2.42	1.67	1.75	0.02
**celiprolol**	1.12	0.05	-0.05	-0.11	0.18	0.001	5.87	0.77	-0.85	-1.71	2.80	0.01
**cephalexin**	0.16	-0.80	-29.83	0.61	-5.68	-0.024	0.00					
**cetrizine**	1.55	0.19	-0.63	0.09	0.20	0.004	1.05	0.02	-0.07	0.01	0.02	0.00
**chloramphenikol**	0.61	-0.21	0.17	0.86	-0.39	-0.002	1.08	0.03	-0.03	-0.13	0.06	0.00
**chlorpromazine**	13.33	1.12	29.20	0.30	1.25	0.035	58.85	1.77	45.94	0.47	1.97	0.06
**chlortalidone**	0.85	-0.07	0.05	0.11	-0.09	-0.001	1.32	0.12	-0.09	-0.19	0.16	0.00
**cimetidine**	0.67	-0.17	0.14	0.29	-1.02	-0.002	0.26	-0.58	0.45	0.98	-3.41	-0.01
**ciprofloxacin**	0.19	-0.72	1.17	0.39	-2.40	-0.010	1.22	0.09	-0.14	-0.05	0.29	0.00
**cisapride**	4.88	0.69	-0.83	0.31	0.71	0.008	57.99	1.76	-2.13	0.78	1.81	0.02
**clarithromycin**	0.82	-0.09	0.04	-0.03	-0.12	0.000	0.10	-1.02	0.43	-0.32	-1.45	-0.01
**clindamycin**	1.34	0.13	-0.11	0.08	0.14	0.001	11.34	1.05	-0.92	0.66	1.13	0.01
**clobazam**	1.01	0.00	-0.04	0.00	0.01	0.000	2.58	0.41	-4.23	-0.29	0.48	0.01
**clonidine**	1.35	0.13	-3.65	0.47	0.43	0.004	2.83	0.45	-12.66	1.61	1.51	0.01
**clorazepate**	3.99	0.60	-0.55	0.22	0.62	0.006	4.88	0.69	-0.63	0.25	0.71	0.01
**clozapine**	6.84	0.84	1.69	0.47	0.84	0.258	50.94	1.71	3.45	0.96	1.72	0.53
**cyproheptadine**	9.7	0.99	9.40	0.85	1.01	0.036	23.79	1.38	13.12	1.19	1.41	0.05
**desloratidine**	5.97	0.78	5.23	11.09	0.92	0.031	50.45	1.70	11.48	24.33	2.02	0.07
**diazepam**	3.57	0.55	22.76	0.18	0.56	0.017	5.08	0.71	29.07	0.23	0.72	0.02
**digoxin**	0.21	-0.68	0.25	-0.25	-2.71	-0.003	3.69	0.57	-0.21	0.21	2.27	0.00
**dihydroergotamine**	0.23	-0.64	0.47	0.30	-0.69	-0.005	0.00					
**diphenhydramin**	1.88	0.27	0.79	0.13	0.28	0.022	3.66	0.56	1.62	0.27	0.57	0.05
**doxazosin**	5.51	0.74	-0.52	0.35	0.76	0.006	0.22	-0.66	0.46	-0.31	-0.67	-0.01
**doxycycline**	1.68	0.23	-0.10	-0.12	0.25	0.001	1.76	0.24	-0.10	-0.13	0.27	0.00
**drotaverine**	1.91	0.28	-1.19	-0.19	0.32	0.006	31.24	1.49	-6.33	-0.99	1.71	0.03
**enalapril**	0.34	-0.47	0.47	-0.20	-0.85	-0.005	0.05	-1.29	1.31	-0.56	-2.35	-0.01
**eplerenone**	0.21	-0.68	0.95	-0.35	-1.36	-0.009	1.54	0.19	-0.26	0.10	0.38	0.00
**escitalopram**	2.84	0.45	-13.67	0.52	0.81	0.013	32.08	1.51	-45.42	1.73	2.69	0.04
**estradiol benzoate**			0.00	0.00	0.00		6.92	0.84	-0.79	0.20	0.88	0.01
**estrone**	11.01	1.04	-1.02	0.36	1.10	0.011	29.40	1.47	-1.44	0.51	1.55	0.02
**famotidine**	0.99	0.00	0.00	0.00	-0.02	0.000	0.26	-0.58	0.18	0.53	-3.33	0.00

**fexofenadine**	1.07	0.03	-0.04	0.01	0.05	0.000	1.08	0.03	-0.04	0.01	0.05	0.00
**fluoxetine**	6.06	0.78	3.78	0.41	0.83	0.037	66.64	1.82	8.82	0.94	1.93	0.09
**flupenthixol**	15.36	1.19	-4.16	0.22	1.25	0.023	51.21	1.71	-5.99	0.32	1.80	0.03
**furosemide**	11.66	1.07	-0.69	-4.44	1.12	0.008	0.28	-0.56	0.36	2.32	-0.59	0.00
**gliclazide**	9.69	0.99	-1.17	0.52	1.05	0.011	0.28	-0.55	0.66	-0.29	-0.59	-0.01
**haloperidol**	4.8	0.68	-6.70	0.52	0.74	0.017	23.74	1.38	-13.53	1.05	1.50	0.03
**hydrocortisone acetate**	0.66	-0.18	0.43	-0.04	-0.19	-0.003	2.82	0.45	-1.07	0.11	0.47	0.01
**hydroxyzine**	3.69	0.57	-20.22	0.46	0.61	0.016	13.82	1.14	-40.67	0.93	1.23	0.03
**ibuprofen**	65.66	1.82	-36.49	0.47	1.91	0.049	0.86	-0.07	1.34	-0.02	-0.07	0.00
**indomethacin**	64.78	1.81	-3.85	-1.27	1.87	0.028	1.08	0.03	-0.07	-0.02	0.03	0.00
**ketoprofen**	24.32	1.39	-4.29	0.54	1.40	0.025	0.20	-0.71	2.19	-0.28	-0.72	-0.01
**ketotifen**	5.83	0.77	-3.33	2.94	1.02	0.016	22.04	1.34	-5.85	5.17	1.79	0.03
**lamotrigine**	1.08	0.03	-0.04	0.02	0.06	0.000	0.71	-0.15	0.16	-0.11	-0.27	0.00
**levocetrizine**	1.77	0.25	-0.82	0.07	0.27	0.005	3.82	0.58	-1.93	0.17	0.64	0.01
**levofloxacin**	3.94	0.60	-0.95	0.96	1.92	0.008	0.90	-0.04	0.07	-0.07	-0.14	0.00
**loperamide**	4.47	0.65	-4.24	0.13	0.67	0.015	0.00					
**loratadine**	10.54	1.02	-7.76	3.41	1.04	0.024	11.76	1.07	-8.12	3.57	1.09	0.03
**lorazepam**	2.21	0.34	-0.78	0.10	0.40	0.006	9.04	0.96	-2.17	0.28	1.10	0.02
**medazepam**	17.86	1.25	4.21	0.29	1.26	0.080	34.65	1.54	5.18	0.36	1.56	0.10
**meloxicam**	63.54	1.80	-1.10	-1.55	1.82	0.013	0.49	-0.31	0.19	0.27	-0.31	0.00
**methyldopa**	0.36	-0.44	0.40	0.30	-2.22	-0.004	0.10	-1.02	0.91	0.70	-5.08	-0.01
**mianserin**	6.39	0.81	1.82	0.86	0.90	0.124	21.72	1.34	3.02	1.42	1.49	0.21
**midazolam**	3.22	0.51	3.55	0.15	0.52	0.020	5.69	0.76	5.28	0.22	0.78	0.03
**mirtazapine**	2.96	0.47	1.99	0.41	0.55	0.024	5.83	0.77	3.23	0.66	0.90	0.04
**montelukast**	0.19	-0.72	1.24	-0.17	-0.73	-0.010	99.10	2.00	-3.44	0.48	2.02	0.03
**naproxen**	90.08	1.95	-9.90	0.65	1.97	0.042	0.31	-0.51	2.59	-0.17	-0.52	-0.01
**nebivolol**	7.49	0.87	-1.49	-0.85	0.89	0.012	0.00					
**olanzapine**	4.83	0.68	-0.86	-0.44	3.91	0.008	20.31	1.31	-1.64	-0.84	7.47	0.02
**oxazepam**	1.65	0.22	-0.49	0.07	0.26	0.004	4.78	0.68	-1.54	0.23	0.80	0.01
**oxybutinin**	4.01	0.60	-2.42	0.19	0.66	0.012	49.68	1.70	-6.80	0.52	1.84	0.03
**pantoprazole**	2.09	0.32	-0.38	-0.17	0.33	0.004	2.19	0.34	-0.41	-0.18	0.35	0.00

**paracetamol**	0.28	-0.55	2.28	0.42	-2.21	-0.011	0.32	-0.49	2.03	0.37	-1.97	-0.01
**paroxetine**	5.96	0.78	-8.76	-1.58	0.82	0.020	115.77	2.06	-23.31	-4.21	2.20	0.05
**pergolide**	0.07	-1.15	7.12	-0.50	-1.28	-0.026	0.00					
**perindopril**	0.49	-0.31	0.31	-0.15	-2.07	-0.003	0.18	-0.76	0.77	-0.37	-5.05	-0.01
**phenytoin**	1.63	0.21	-0.55	0.29	0.24	0.004	1.78	0.25	-0.65	0.34	0.28	0.00
**pindolol**	1.65	0.22	-0.59	-0.18	0.54	0.004	6.09	0.78	-2.12	-0.64	1.96	0.01
**piroxicam**	32.92	1.52	-1.29	1.42	1.53	0.014	0.16	-0.79	0.67	-0.74	-0.80	-0.01
**prednizolone**	0.52	-0.28	0.29	-0.14	-0.32	-0.003	2.84	0.45	-0.47	0.23	0.50	0.00
**progesterone**	5.7	0.76	-3.83	0.24	0.78	0.016	34.99	1.54	-7.82	0.49	1.58	0.03
**promazine**	8.9	0.95	24.65	0.29	1.01	0.030	0.10	-1.02	-26.48	-0.31	-1.08	-0.03
**promethazine**	5.35	0.73	18.91	-3.64	0.78	0.023	14.96	1.17	30.50	-5.87	1.26	0.04
**propafenone**	3.23	0.51	-1.31	0.27	0.52	0.009	66.74	1.82	-4.68	0.98	1.88	0.03
**propranolol**	3.48	0.54	-4.64	0.19	0.60	0.013	28.09	1.45	-12.40	0.52	1.61	0.03
**quetiapine**	2.48	0.39	-0.63	0.14	0.48	0.005	17.02	1.23	-1.95	0.43	1.48	0.02
**quinapril**	3.1	0.49	-0.50	0.29	0.51	0.005	1.22	0.09	-0.09	0.05	0.09	0.00
**risperidone**	2.11	0.32	-0.73	0.51	0.37	0.005	4.83	0.68	-1.54	1.09	0.78	0.01
**rizatriptan**	2.06	0.31	-0.79	-1.16	2.24	0.005	2.53	0.40	-1.01	-1.49	2.88	0.01
**rosuvastatin**	1.96	0.29	-0.17	0.25	0.33	0.002	0.96	-0.02	0.01	-0.02	-0.02	0.00
**roxitromicin**	1.03	0.01	0.00	0.00	0.01	0.000	0.00					
**rupatadine**	12.38	1.09	13.22	0.67	1.11	0.038	9.29	0.97	11.71	0.59	0.98	0.03
**sertraline**	13.19	1.12	3.16	0.67	1.14	0.093	42.89	1.63	4.60	0.97	1.67	0.14
**sildenafil**	1.62	0.21	-0.17	-0.14	0.22	0.002	14.26	1.15	-0.96	-0.79	1.20	0.01
**simvastatin**	8.72	0.94	-1.52	0.21	0.99	0.013	13.21	1.12	-1.81	0.25	1.18	0.02
**spironolactone**	0.82	-0.09	0.10	-0.03	-0.10	-0.001	13.38	1.13	-1.37	0.34	1.25	0.01
**sulpiride**	1.45	0.16	-0.13	0.34	0.40	0.001	1.66	0.22	-0.18	0.46	0.55	0.00
**telmisartan**	22.51	1.35	-2.93	0.18	1.36	0.021	4.68	0.67	-1.45	0.09	0.67	0.01
**temazepam**	1.26	0.10	-0.33	0.03	0.10	0.002	6.74	0.83	-2.77	0.26	0.86	0.02
**theophylline**	0.22	-0.66	1.17	0.50	-1.64	-0.009	0.12	-0.94	1.67	0.72	-2.35	-0.01
**thioridazine**	18.57	1.27	-3.46	0.30	1.34	0.022	47.61	1.68	-4.58	0.40	1.77	0.03
**tolterodine**	2.9	0.46	2.70	0.14	0.48	0.020	0.21	-0.68	-3.95	-0.21	-0.70	-0.03
**trazodone**	2.22	0.35	-2.64	0.12	0.38	0.008	9.30	0.97	-7.38	0.35	1.05	0.02

**tropicamid**	0.15	-0.82	2.68	82.39	-1.83	-0.015	1.06	0.03	-0.09	-2.68	0.06	0.00
**valproic acid**	4.46	0.65	-13.04	0.25	0.76	0.017	0.08	-1.09	21.90	-0.42	-1.28	-0.03
**valsartan**	48.78	1.69	-1.35	0.44	1.77	0.015	0.08	-1.10	0.88	-0.28	-1.15	-0.01
**venlafaxine**	1.05	0.02	0.89	0.01	0.08	0.001	8.25	0.92	38.52	0.64	3.40	0.03
**verapamil**	2.22	0.35	-0.73	0.61	0.38	0.005	11.97	1.08	-2.26	1.89	1.20	0.02
**zolmitriptane**	1.7	0.23	-0.62	-0.23	0.92	0.004	3.30	0.52	-1.40	-0.51	2.07	0.01
**zolpidem**	1.77	0.25	-4.53	0.29	0.27	0.007	2.60	0.42	-7.58	0.48	0.45	0.01
**zopiclone**	0.76	-0.12	0.13	-0.05	-0.26	-0.001	0.91	-0.04	0.04	-0.02	-0.09	0.00
**zuclopenthixol**	16.18	1.21	-4.24	0.24	1.23	0.023	48.73	1.69	-5.92	0.34	1.71	0.03

Table S6. Biological properties for APIs from the external group (n = 38). An external API group (n = 38) was assembled at a later time than previous one (n = 129).

API	B1	B2	B3	BB_{vivo}	CNS+/-	LactMed	LLL H	log U/D 7.2	M/P	PB	PhCharge
**acetazolamid**	-1.60	-1.29	0.52		1	1	2	-0.24	0.28	0.98	-1
**amitriptyline**	0.76	0.50	-0.45	0.89	1	1	2	-1.98	0.92	0.95	1
**bupivacaine**	0.16	0.03	1.25		1	1	2	-0.93	0.37	0.95	1
**chloroquine**	0.39	0.10	0.89		1	1	2	-3.27	2.65	0.55	2
**citalopram**	0.13	-0.03	-0.23		1	2	2	-2.37	1.03	0.80	1
**clomipramine**	0.79	0.44	-0.28		1	1	2	-2.26	1.13	0.98	1
**colchicine**	-0.93	-0.78	-1.99		1	2	4		0.95	0.39	0
**diltiazem**	-0.02	-0.40	-1.22		1	1	3	-1.74	0.99	0.75	1
**doxepin**	0.54	0.35	-0.18		1	1	5	-2.20	1.37	0.76	1
**duloxetine**	0.56	0.21	-0.65		1	1	3	-2.82	0.78	0.90	1
**eletriptan**	-0.19	-0.30	-0.84		1	1	3	-3.15	0.25	0.85	1
**ethambutol**	-0.86	-0.49	-0.52		0	1	2	-2.39	1.00	0.25	1
**ethanol**	-0.19	0.22	-0.67	-0.16	1	2	3		0.95	0.00	0
**fluvoxamine**	-0.14	-0.36	0.68		1	1	2	-2.19	1.33	0.79	1
**gabapentin**	-0.63	-0.47	-0.19		1	1	2	-0.29	1.00	0.03	0
**gentamycin**	-3.13	-2.65	-2.40		0	1	2	-2.57	0.36	0.15	5
**hydrochlorotiazide**	-1.62	-1.35	-2.29		0	1	2	-1.75	0.38	0.68	0
**itraconazole**	-0.66	-1.07	3.68		0	1	2	-0.73	1.14	1.00	0
**ketorolac**	-0.33	-0.40	0.23		1	1	2	-2.91	0.03	0.99	-1
**levetiracetam**	-0.93	-0.47	-1.02		1	1	3		1.00	0.10	0
**lincomycin**	-1.62	-1.47	-0.43		0		2	-1.58	0.15	0.70	1
**mesalazine**	-0.99	-0.79	-1.55		0	1	3	-5.30	2.69	0.43	-1
**minoxidil**	-1.00	-0.95	-0.18		1		3	-1.66	0.82	0.00	0
**nitrendipine**	-0.92	-1.22	-1.09		0	1	2	-4.41	0.30	0.99	0
**ofloxacin**	-0.66	-0.63	-1.97		0	1	2	-2.01	1.26	0.32	-1

**oxcarbamazepine**	-0.55	-0.47	-0.87		1		3	-6.53	0.50	0.40	0
**pefloxacin**	-0.52	-0.48	-1.44		0			-7.04	0.96	0.25	-1
**pregabalin**	-0.63	-0.47	-0.22		1	1	3	-0.57	0.55	0.00	0
**primidone**	-0.60	-0.38	-0.31		1	2	3	-5.06	0.72	0.70	0
**propylthiouracil**	-0.29	-0.11	-0.31		1	1	2	-0.43	0.17	0.82	0
**quinine**	-0.11	-0.18	-0.07		1	1	2	-2.08	0.23	0.70	1
**rifampicin**	-2.81	-2.98	-0.67		0	1	2	-2.24	0.40	0.89	1
**rimantadine**	0.26	0.13	0.43		1		3	-3.56	2.00	0.40	1
**timolol**	-0.85	-0.73	-0.06		0	1	2	-2.15	0.80	0.10	1
**tinidazole**	-1.35	-1.02	-0.65		1	1	3	-4.90	1.14	0.12	0
**tramadol**	0.01	0.02	-0.67		1	1	2	-2.41	2.30	0.20	1
**trimethoprim**	-1.33	-1.14	-1.90		1	1	2	-0.30	1.25	0.44	1
**warfarin**	-0.33	-0.47	-0.44		1	0	2	-2.70	0.00	0.99	-1

Table S7. Physicochemical properties for APIs from the external group (n = 38).

API	a/b/n code[1]	DM	eH	eH-eL	eL[2]	HA	HD	log D	log P	MW[3]	pKa	PSA[4]	Sa[3]	V[3]
**acetazolamid**	0	0.89	-9.06	-9.13	0.69	5	2	-0.55	-0.26	2.22	7.44	11.50	4.69	2.9
**amitriptyline**	1	6.42	-9.9	-8.20	-17.01	1	0	3.15	4.41	2.77	9.18	0.32	3.61	1.56
**bupivacaine**	0	3.02	-9.03	-9.37	3.37	2	1	2.80	3.31	2.88	8.13	3.23	4.78	3.02
**chloroquine**	1	4.4	-8.6	-8.03	-5.71	3	1	1.87	4.41	3.20	10.47	2.82	5.94	3.11
**citalopram**	1	2.83	-7.98	-8.86	8.83	3	0	0.39	3.48	3.24	9.57	3.63	5.88	3.14
**clomipramine**	1	1.53	-8.49	-8.65	1.56	2	0	3.50	4.94	3.15	9.46	0.65	5.11	3.05
**colchicine**	0	5.95	-9.11	-8.52	-5.91	6	1	0.92	1.07	3.99		8.31	5.47	3.61
**diltiazem**	1	2.43	-8.71	-8.52	-1.86	4	0	2.10	4.73	4.15	8.94	5.91	6.17	3.96
**doxepin**	1	0.31	-9.11	-9.03	-0.81	2	0	2.08	3.84	2.79	9.40	1.25	4.69	2.81
**duloxetine**	1	0.96	-9.01	-8.63	-3.82	2	1	1.24	4.81	2.97	10.02	2.13	4.56	2.84
**eletriptan**	1	4.88	-8.5	-7.90	-5.96	3	1	0.36	2.98	3.83	10.35	5.32	4.69	3.57
**ethambutol**	1	1.11	-9.11	-11.45	23.39	4	4	-2.21	-0.29	2.04	9.59	6.45	4.95	2.18
**ethanol**	0					1	1	-0.19	-0.18	0.46		2.02		
**fluvoxamine**	1	3.9	-9.69	-8.87	-8.25	4	1	1.15	3.71	3.18	9.39	5.68	5.89	2.79
**gabapentin**	0	1.56	-9.35	-10.19	8.36	3	2	-1.31	1.08	1.71	7.50	6.33	2.38	1.7
**gentamycin**	1	4.31	-9.34	-11.20	18.62	12	8	-7.81	-2.04	4.78	9.77	19.97	4.71	4.25
**hydrochlorotiazide**	0	7.27	-9.64	-8.46	-11.83	5	3	-0.09	-0.02	2.98	8.95	11.84	3.85	2.01
**itraconazole**	0	6.11	-8.42	-7.65	-7.7	9	0	4.26	4.53	6.73	6.47	10.08	8.43	6.15
**ketorolac**	-1	2.14	-9.19	-8.83	-3.63	3	1	-0.95	2.68	2.55	4.29	5.93	3.87	2.39
**levetiracetam**	0	5.71	-9.8	-10.45	6.49	2	1	-0.67	-0.88	1.70		6.34	3.09	1.62
**lincomycin**	1	3.92	-8.5	-8.50	0.04	8	5	-0.48	0.72	4.07	8.78	12.60	6.35	3.89
**mesalazine**	-1	3.8	-8.18	-8.06	-1.23	4	3	-2.61	0.74	1.53	1.90	8.36	2.49	1.28
**minoxidil**	0	4.2	-8.07	-8.10	0.29	5	2	0.65	1.62	2.09	5.54	9.36	3.16	1.96
**nitrendipine**	0	6.92	-9.09	-8.00	-10.94	5	1	3.50	3.81	3.60	2.79	11.05	5.3	2.98
**ofloxacin**	-1	4.68	-8.72	-7.82	-8.97	7	1	-0.65	1.85	3.61	5.19	7.33	4.74	3.07
**oxcarbamazepine**	0	3.46	-9.31	-8.84	-4.73	2	1	1.25	1.66	2.52	13.73	6.34	3.02	2.26
**pefloxacin**	-1	9.41	-9.01	-7.97	-10.45	6	1	0.52	1.92	3.33	0.16	6.41	4.66	2.91
**pregabalin**	0	1.67	-9.53	-10.46	9.29	3	2	-1.38	1.09	1.59	7.77	6.33	3.54	1.65

**primidone**	0	2.84	-9.78	-9.77	-0.15	2	2	0.40	0.83	2.18	12.26	5.82	3.19	2.02
**propylthiouracil**	0	5.57	-9.2	-7.93	-12.71	1	2	1.18	1.15	1.70	7.63	4.11	3.32	1.51
**quinine**	1	1.73	-9.55	-9.54	-0.14	4	1	1.58	2.82	3.24	9.28	4.56	4.22	3.06
**rifampicin**	-1					14	6	-0.46	2.05	8.23	4.96	22.02		
**rimantadine**	1	1.22	-9.33	-12.28	29.54	1	1	0.08	3.31	1.79	10.76	2.60	2.43	1.91
**timolol**	1	3.06	-9.17	-8.05	-11.24	7	2	-1.39	1.28	3.16	9.35	7.97	4.95	2.88
**tinidazole**	0	2.34	-10.52	-9.24	-12.82	5	0	-0.27	-0.29	2.47	2.30	9.78	4.41	2
**tramadol**	1					3	1	0.36	2.32	2.63	9.61	3.27		
**trimethoprim**	0	2.09	-8.8	-8.68	-1.22	7	2	0.58	0.59	2.90	6.90	10.55	4.14	2.63
**warfarin**	-1	4.12	-7.38	-6.05	-13.35	3	1	-0.90	3.13	3.08	4.50	6.36	4.01	2.76

1 – code describing the acid-base properties: (-1) - acids; (0) - neutral drugs; (1) - bases

2 – value multiplied by 10

3 – value divided by 100

4 – value divided by 10

Table S8. Chromatographic data and their derivatives obtained from the NP TLC and RP TLC experiment for the external group of APIs (n = 38).

API	C_{NP}*	NP**	NP/C	NP/PSA	C_{RP}*	RP**	RP/C	RP/PSA	NP/B2	RP/B2	NP/PB	RP/PB	NP/logP	RP/logP
**acetazolamid**	0.94	0.94	1.00	0.817	0.97	0.96	0.99	0.834	-0.727	-0.742	0.959	0.980	-3.615	-3.692
**amitriptyline**	0.32	0.31	0.97	9.568	0.67	0.63	0.94	19.444	0.626	1.272	0.326	0.663	0.070	0.143
**bupivacaine**	0.67	0.67	1.00	2.072	0.74	0.75	1.01	2.319	22.666	25.372	0.705	0.789	0.202	0.227
**chloroquine**	0.17	0.17	1.00	0.604	0.40	0.30	0.75	1.065	1.763	3.111	0.309	0.545	0.039	0.068
**citalopram**	0.30	0.29	0.97	0.800	0.62	0.60	0.97	1.655	-8.745	-18.094	0.363	0.750	0.083	0.172
**clomipramine**	0.33	0.32	0.97	4.938	0.59	0.59	1.00	9.105	0.722	1.331	0.327	0.602	0.065	0.119
**colchicine**	0.79	0.77	0.97	0.927	0.91	0.91	1.00	1.095	-0.984	-1.163	1.974	2.333	0.720	0.850
**diltiazem**	0.49	0.49	1.00	0.829	0.76	0.72	0.95	1.219	-1.230	-1.808	0.653	0.960	0.104	0.152
**doxepin**	0.30	0.31	1.03	2.486	0.58	0.54	0.93	4.330	0.892	1.554	0.411	0.715	0.081	0.141
**duloxetine**	0.32	0.33	1.03	1.552	0.57	0.53	0.93	2.493	1.595	2.562	0.367	0.589	0.069	0.110
**eletriptan**	0.29	0.28	0.97	0.527	0.62	0.62	1.00	1.166	-0.922	-2.041	0.329	0.729	0.094	0.208
**ethambutol**	0.19	0.21	1.11	0.325	0.97	0.95	0.98	1.472	-0.433	-1.957	0.840	3.800	-0.724	-3.276
**ethanol**														
**fluvoxamine**	0.34	0.32	0.94	0.563	0.62	0.62	1.00	1.091	-0.883	-1.711	0.408	0.790	0.086	0.167
**gabapentin**	0.91	0.91	1.00	1.437	0.66	0.66	1.00	1.042	-1.952	-1.416	30.333	22.000	0.843	0.611
**gentamycin**	0.96	0.94	0.98	0.471	0.91	0.90	0.99	0.451	-0.355	-0.340	6.267	6.000	-0.461	-0.441
**hydrochlorotiazide**	0.96	0.96	1.00	0.811	0.97	0.96	0.99	0.811	-0.713	-0.713	1.414	1.414	-48.000	-48.000
**itraconazole**	0.96	0.97	1.01	0.962	0.86	0.84	0.98	0.833	-0.910	-0.788	0.972	0.842	0.214	0.185
**ketorolac**	0.92	0.91	0.99	1.535	0.96	0.96	1.00	1.619	-2.265	-2.389	0.919	0.970	0.340	0.358
**levetiracetam**	0.92	0.92	1.00	1.451	0.92	0.92	1.00	1.451	-1.968	-1.968	9.200	9.200	-1.045	-1.045
**lincomycin**	0.91	0.91	1.00	0.722	0.94	0.94	1.00	0.746	-0.620	-0.640	1.300	1.343	1.264	1.306
**mesalazine**	0.94	0.93	0.99	1.113	0.99	0.97	0.98	1.161	-1.178	-1.228	2.163	2.256	1.257	1.311
**minoxidil**	0.43	0.42	0.98	0.449	0.80	0.82	1.03	0.876	-0.442	-0.862			0.259	0.506
**nitrendipine**	0.97	0.97	1.00	0.878	0.86	0.86	1.00	0.779	-0.795	-0.705	0.980	0.869	0.255	0.226
**ofloxacin**	0.24	0.23	0.96	0.314	0.54	0.50	0.93	0.682	-0.367	-0.799	0.719	1.563	0.124	0.270
**oxcarbamazepine**	0.91	0.90	0.99	1.420	0.91	0.91	1.00	1.435	-1.926	-1.947	2.250	2.275	0.542	0.548
**pefloxacin**	0.30	0.29	0.97	0.452	0.76	0.70	0.92	1.092	-0.606	-1.463	1.160	2.800	0.151	0.365
**pregabalin**	0.76	0.76	1.00	1.200	0.41	0.41	1.00	0.648	-1.630	-0.880			0.697	0.376

**primidone**	0.90	0.93	1.03	1.598	0.93	0.97	1.04	1.667	-2.421	-2.525	1.329	1.386	1.120	1.169
**propylthiouracil**	0.94	0.94	1.00	2.285	0.93	0.93	1.00	2.261	-8.462	-8.372	1.146	1.134	0.817	0.809
**quinine**	0.30	0.29	0.97	0.636	0.66	0.64	0.97	1.404	-1.590	-3.508	0.414	0.914	0.103	0.227
**rifampicin**	0.73	0.77	1.05	0.350	0.95	0.94	0.99	0.427	-0.259	-0.316	0.865	1.056	0.376	0.459
**rimantadine**	0.92	0.92	1.00	3.536	0.95	0.95	1.00	3.651	7.040	7.270	2.300	2.375	0.278	0.287
**timolol**	0.34	0.32	0.94	0.401	0.97	0.97	1.00	1.216	-0.439	-1.331	3.200	9.700	0.250	0.758
**tinidazole**	0.89	0.88	0.99	0.900	0.94	0.93	0.99	0.951	-0.865	-0.914	7.333	7.750	-3.034	-3.207
**tramadol**	0.31	0.31	1.00	0.948	0.63	0.64	1.02	1.957	13.025	26.891	1.550	3.200	0.134	0.276
**trimethoprim**	0.57	0.59	1.04	0.559	0.84	0.83	0.99	0.787	-0.517	-0.727	1.341	1.886	1.000	1.407
**warfarin**	0.99	0.99	1.00	1.557	0.87	0.83	0.95	1.305	-2.104	-1.764	1.000	0.838	0.316	0.265

* R_f value from the non-impregnated NP or RP plate (control)

** R_f value from the NP or RP plate impregnated with 2 mg/mL BSA solution

Table S9. Chromatographic data from $HPLC_{HSA}$ and $HPLC_{IAM}$ experiments and their derivatives obtained for 38 external APIs.

API	k_{HSA}	log k_{HSA}	log k_{HSA}/B2	log k_{HSA}/PB	log k_{HSA}/log P	k_{IAM}	log k_{IAM}
**acetazolamid**	0.74	-0.13	-0.575	0.760	-2.863	2.90	0.46
**amitriptyline**	5.87	0.77	11.854	6.178	1.331	1.56	0.19
**bupivacaine**	1.18	0.07	40.084	1.247	0.358	3.02	0.48
**chloroquine**	18.05	1.26	187.127	32.812	4.092	3.11	0.49
**citalopram**	2.67	0.43	-80.418	3.333	0.766	3.14	0.50
**clomipramine**	9.09	0.96	20.510	9.278	1.841	3.05	0.48
**colchicine**	0.28	-0.55	-0.362	0.726	0.265	3.61	0.56
**diltiazem**	1.74	0.24	-4.362	2.316	0.367	3.96	0.60
**doxepin**	4.25	0.63	12.220	5.624	1.106	2.81	0.45
**duloxetine**	8.16	0.91	39.435	9.063	1.696	2.84	0.45
**eletriptan**	3.78	0.58	-12.429	4.441	1.267	3.57	0.55
**ethambutol**	1.73	0.24	-3.557	6.906	-5.953	2.18	0.34
**ethanol**	0.02	-1.76	0.078		-0.097	0.01	-2.09
**fluvoxamine**	0.25	-0.60	-0.696	0.321	0.068	2.79	0.45
**gabapentin**	0.15	-0.84	-0.312	4.843	0.135	1.70	0.23
**gentamycin**	1.50	0.18	-0.568	10.028	-0.737	4.25	0.63
**hydrochlorotiazide**	0.61	-0.22	-0.452	0.897	-30.451	2.01	0.30
**itraconazole**	9.65	0.98	-9.055	9.668	2.130	6.15	0.79
**ketorolac**	9.65	0.98	-24.017	9.747	3.601	2.39	0.38
**levetiracetam**	0.07	-1.16	-0.149	0.696	-0.079	1.62	0.21
**lincomycin**	0.82	-0.08	-0.560	1.175	1.142	3.89	0.59
**mesalazine**	0.18	-0.73	-0.234	0.430	0.250	1.28	0.11
**minoxidil**	0.40	-0.40	-0.422		0.248	1.96	0.29
**nitrendipine**	3.55	0.55	-2.906	3.581	0.931	2.98	0.47
**ofloxacin**	6.62	0.82	-10.566	20.673	3.576	3.07	0.49
**oxcarbamazepine**	0.40	-0.40	-0.846	0.988	0.238	2.26	0.35
**pefloxacin**	12.43	1.09	-25.980	49.720	6.474	2.91	0.46
**pregabalin**	0.14	-0.87	-0.291		0.124	1.65	0.22

**primidone**	0.22	-0.66	-0.569	0.312	0.263	2.02	0.31
**propylthiouracil**	0.33	-0.48	-2.950	0.400	0.285	1.51	0.18
**quinine**	2.59	0.41	-14.194	3.699	0.918	3.06	0.49
**rifampicin**	0.03	-1.53	-0.010	0.034	0.015	8.37	0.92
**rimantadine**	0.16	-0.80	1.222	0.399	0.048	1.91	0.28
**timolol**	0.13	-0.88	-0.179	1.304	0.102	2.88	0.46
**tinidazole**	0.12	-0.92	-0.118	0.997	-0.413	2.00	0.30
**tramadol**	1.08	0.03	45.221	5.381	0.464	3.90	0.59
**trimethoprim**	0.85	-0.07	-0.749	1.943	1.449	2.63	0.42
**warfarin**	13.86	1.14	-29.454	14.001	4.428	2.76	0.44
